# Molecular and functional changes in neutrophilic granulocytes induced by nicotine: a systematic review and critical evaluation

**DOI:** 10.3389/fimmu.2023.1281685

**Published:** 2023-11-23

**Authors:** Theresa-Charlotte Brembach, Robert Sabat, Katrin Witte, Tanja Schwerdtle, Kerstin Wolk

**Affiliations:** ^1^ Psoriasis Research and Treatment Center, Charité – Universitätsmedizin Berlin, Berlin, Germany; ^2^ Department of Food Chemistry, Institute of Nutritional Science, University of Potsdam, Nuthetal, Germany; ^3^ German Federal Institute for Risk Assessment (BfR), Berlin, Germany

**Keywords:** PMN, smoking, hidradenitis suppurativa, palmoplantar pustulosis, psoriasis, inflammation, antimicrobial defense

## Abstract

**Background:**

Over 1.1 billion people smoke worldwide. The alkaloid nicotine is a prominent and addictive component of tobacco. In addition to tumors and cardiovascular disorders, tobacco consumption is associated with a variety of chronic-inflammatory diseases. Although neutrophilic granulocytes (neutrophils) play a role in the pathogenesis of many of these diseases, the impact of nicotine on neutrophils has not been systematically reviewed so far.

**Objectives:**

The aim of this systematic review was to evaluate the direct influence of nicotine on human neutrophil functions, specifically on cell death/damage, apoptosis, chemotaxis, general motility, adhesion molecule expression, eicosanoid synthesis, cytokine/chemokine expression, formation of neutrophil extracellular traps (NETs), phagocytosis, generation of reactive oxygen species (ROS), net antimicrobial activity, and enzyme release.

**Material and methods:**

This review was conducted according to the PRISMA guidelines. A literature search was performed in the databases NCBI Pubmed^®^ and Web of Science™ in February 2023. Inclusion criteria comprised English written research articles, showing *in vitro* studies on the direct impact of nicotine on specified human neutrophil functions.

**Results:**

Of the 532 originally identified articles, data from 34 articles were finally compiled after several evaluation steps. The considered studies highly varied in methodological aspects. While at high concentrations (>3 mmol/l) nicotine started to be cytotoxic to neutrophils, concentrations typically achieved in blood of smokers (in the nmol/l range) applied for long exposure times (24-72h) supported the survival of neutrophils. Smoking-relevant nicotine concentrations also increased the chemotaxis of neutrophils towards several chemoattractants, elevated their production of elastase, lipocalin-2, CXCL8, leukotriene B4 and prostaglandin E2, and reduced their integrin expression. Moreover, while nicotine impaired the neutrophil phagocytotic and anti-microbial activity, a range of studies demonstrated increased NET formation. However, conflicting effects were found on ROS generation, selectin expression and release of β-glucuronidase and myeloperoxidase.

**Conclusion:**

Nicotine seems to support the presence in the tissue and the inflammatory and selected tissue-damaging activity of neutrophils and reduces their antimicrobial functions, suggesting a direct contribution of nicotine to the pathogenesis of chronic-inflammatory diseases via influencing the neutrophil biology.

## 1 Introduction

Nicotine (3-(1-methyl-pyrrolidinyl)-pyridine) is a naturally occurring alkaloid, which is mainly produced by the nightshade family of plants, particularly the *Nicotiana* ssp. (tobacco plants). Stored in the leaves, it serves as a botanical insecticide for the tobacco plant (1, 2). Given their stimulating and addictive effect on humans, tobacco leaves have been consumed by humans for many centuries. Today they are used for smoking in cigarettes, cigars, pipes, hookahs, and e-cigarettes, in smokeless forms in mouth and oral tobacco, in pharmaceutical products for smoking cessation such as plasters and gums as well as in nicotine medication (3). Currently, there are over 17% tobacco users in the USA (4) and over 1.1 billion smokers worldwide (5). During tobacco consummation, nicotine can be absorbed buccally, in the upper airways, and in the lungs. The average nicotine blood levels found in smokers vary between ~0.1 - 500 nmol/l right after smoking (6–11). Local nicotine levels may exceed those found in the blood of smokers. In fact, much higher nicotine concentrations were found in the sputum of smokers (~30 µmol/l) (12) and in vapers (~45 µmol/l) (13). Chronic exposure to nicotine leads to its accumulation in the tissue (14, 15). As the main psychoactive component in tobacco and e-cigarettes, the pharmacological mode of action of nicotine on the central and the peripheral nervous system is well investigated (16). Nicotine acts as a ligand on the nicotinic acetylcholine receptors (nAChRs). The nAChRs are a group of pentameric cation channels, which consist of a varying combination of α_2-7_, α_9-10_ and β_2-4_ subunits (17). Apart from their localization in the brain and peripheral nervous system, they are found on several non-neuronal cells including neutrophilic granulocytes (in the following referred to as neutrophils) (18–20). In addition to its agonistic action on nAChRs, nicotine can be absorbed into the cells *via* a facilitative transporter or proton/organic cation antiporter (21).

Tobacco consumption is a well-known risk factor for the development and persistence of serious diseases such as cancers and cardiovascular disorders (22, 23). The association of nicotine with chronic-inflammatory diseases (CID) of the lung, the joints, and the skin, has been less researched (24–29). However, ~40% of patients with rheumatoid arthritis are current smokers and 65% of them reported they had previously smoked cigarettes (30). Furthermore, over 80% of patients with hidradenitis suppurativa (HS), a chronic inflammatory disease affecting intertriginous skin areas, are smokers or ex-smokers (31, 32). Other dermatological studies stated that over 70% of patients with palmoplantar pustulosis (PPP) were smokers (28) and 65% of psoriasis patients were current or ex-smokers and the disease severity of psoriasis is associated with smoking intensity (33). Cutaneous CIDs often concern already young adults (34), strongly impair the quality of life of those affected (35, 36) and cause considerable loss of national gross value added (37).

In many of these diseases, neutrophils play important pathogenetic roles. For example, the neutrophils that are abundant in the dermis of skin lesions in HS release extracellular matrix-degrading enzymes that disrupt the physiological structure of the skin (38, 39) and are a source of mediators that support the cutaneous persistence and activation of further immune cells (40, 41). Furthermore, neutrophils form the so-called Munro’s abscesses in the (sub-)corneal layer of the psoriatic epidermis (42). In PPP neutrophils produce high levels of IL-19, a cytokine that supports the further recruitment of these cells into the skin, by triggering keratinocytes to express the chemokine CXCL6 (43). Further effector functions of neutrophils that contribute to inflammation and tissue damage in these skin diseases include reactive oxygen species (ROS) generation and formation of neutrophil extracellular traps (NET) (44).

The association of smoking with neutrophil-driven CID led to the question of whether nicotine exerts direct molecular and functional changes in neutrophils. We therefore performed a systematic review of available data on the direct influence of nicotine on primary human neutrophils.

## 2 Method

This systematic review was conducted according to the Preferred Reporting Items for Systematic Review and Meta-Analysis (PRISMA; http://www.prisma-statement.org/). As there is no system for registration for the non-clinical systematic reviews, the review protocol can be obtained by contacting the corresponding author.

The question to be investigated was: Does nicotine exposure in *in vitro* conditions lead to changes in the following characteristics and functions of human neutrophil: cell death/damage, apoptosis, chemotaxis, general motility, adhesion molecule expression, eicosanoid synthesis, cytokine/chemokine expression, formation of neutrophil extracellular traps (NETs), phagocytosis, generation of reactive oxygen species (ROS), net antimicrobial activity, and enzyme release. This research question was constructed according to the PICOS framework (**Table 1**).

**Table 1:** Research question presented base on the PICOS framework.

PICOS	
Experimental model (Population)	human primary neutrophils
Indicator (**I**ntervention)	nicotine exposure
Control (**C**omparison)	no nicotine exposure
**O**utcome	Direct effect on neutrophils specifically on cell death/damage, apoptosis, chemotaxis, general motility, adhesion molecule expression, eicosanoid synthesis, cytokine/chemokine expression, formation of NETs, phagocytosis, generation of ROS, net antimicrobial activity, and degrading enzyme release
**S**tudy design (context)	*In vitro*, experimental

### 2.1 Literature search

The primary literature search was done using the databases Web of Science™ and NCBI Pubmed^®^ in February 2023. TCB and KWi independently searched the two databases using the search term: nicotine AND (neutrophil* OR granulocyte* OR PMN). All research articles listed in these databases published or accepted for publication until January 31, 2023, were considered.

### 2.2 Eligibility criteria

All articles, in which the influence of nicotine on primary human neutrophils has been studied, were considered. The articles had to be published or accepted for publication and had to be written in English. Articles describing literature reviews, meta-analyses, clinical trials, cohort studies, case-control studies, case series, cross-sectional studies, or case-reports and meeting abstracts were *a priori* excluded. Research articles describing the investigation of nicotine effects on cell lines (e.g. HL-60) or on neutrophils derived from species other than humans were also excluded. After full-text reading, only articles studying the effects of nicotine on the neutrophil characteristics/functions listed in **Table 1** were included.

### 2.3 Study selection

Using the previously determined criteria, TCB and KWi screened both the titles and abstracts of the articles. KWo independently made the decision to include or not articles for which there was no consensus in TCB’s and KWi’s selection. Selected articles were subjected to full-text reading by TCB. Articles that did not fulfil the criteria after full-text reading were excluded after discussion between TCB, KWo and RS. Final decision on inclusion of results was made after discussion between TCB and KWo.

### 2.4 Data extraction

TCB extracted the relevant data in a tabular format (investigated neutrophil function, assays used, investigated cells, cell purity, nicotine exposure time, nicotine compound/dosage form, tested concentration, effective concentration/IC50/EC50, level of significance and number of experiments). Authors were contacted for missing or contradictory published data.

## 3 Results

**Figure 1:**
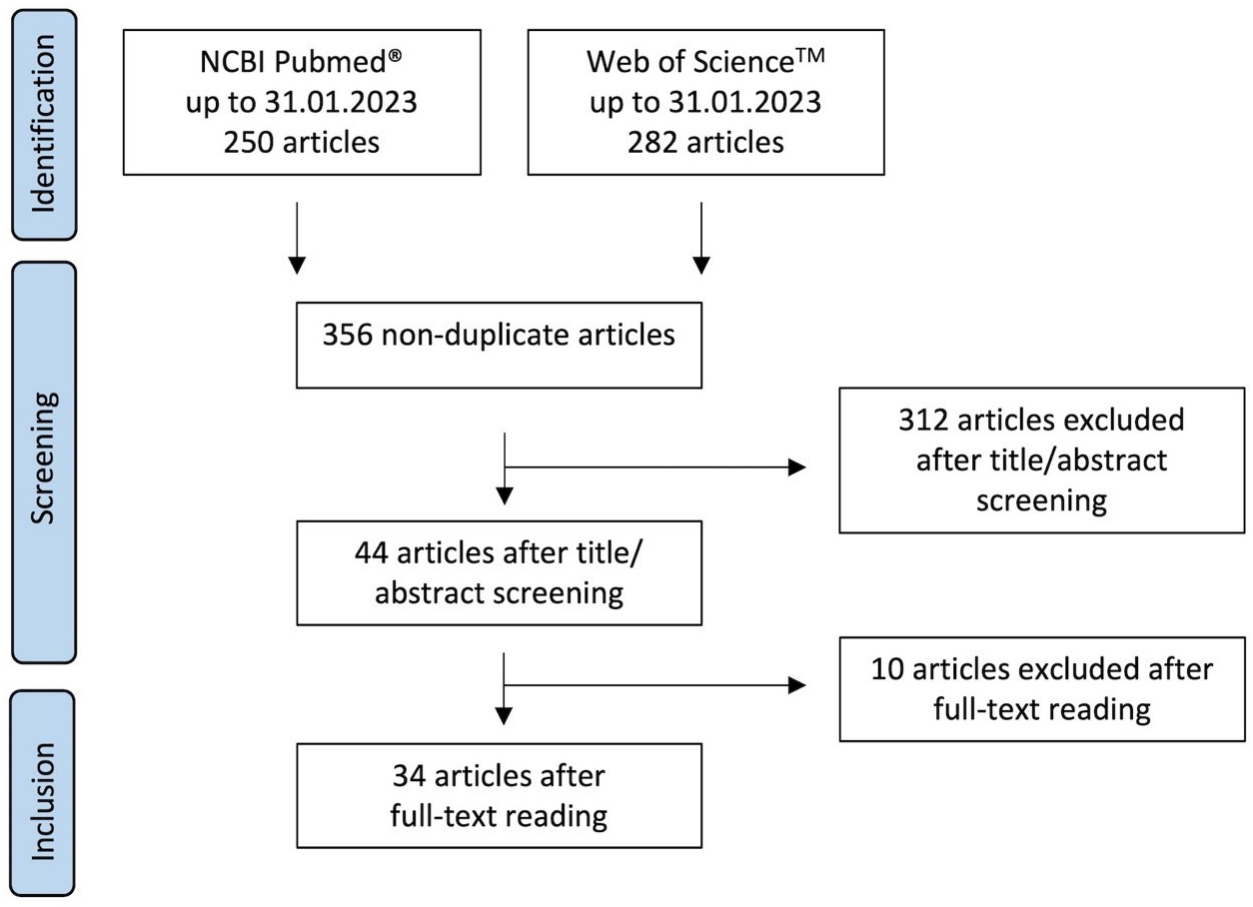
Flow diagram showing the article selection process.

The article selection process is summarized in **Figure 1**. The literature research identified 532 articles in the two databases. 356 non-duplicate articles were subjected to title/abstract screening, of which 44 articles met the criteria for subsequent full-text reading. Ten articles were excluded after full-text reading, as they did not meet the inclusion criteria: In three of them non-human neutrophils were used and in further three the nicotine/epibatidine binding sites and/or the nicotine sensitivity of neutrophils were evaluated. The experiments of one article were based on a cell-free system. Another article described the investigation of neutrophils isolated from participants treated with nicotine-containing gum. Moreover, there was one article showing experiments that lacked untreated controls and one article that didn´t present the data. Thus, 34 articles were included for data extraction (13, 19, 45–76). We have not evaluated or assessed the data for their scientific plausibility. Therefore, no data were excluded because they did not appear scientifically plausible to us.

The results grouped according to the twelve biological functions are displayed in **Tables 2**–**13**. The investigated neutrophil function, assays used, nicotine exposure time, tested nicotine concentration, effective nicotine concentration and IC50/EC50 (if specified), level of significance, and number of experiments are presented. As far as specified by the authors, examined neutrophils were obtained from healthy non-smokers and their purity was above 90%, except for one study with an indicated purity of ~80% (48). The used compound was either (-)-nicotine or nicotine bitartrate.

**Table 2:** Influence of nicotine on general cell death/damage.

Effect = ↓ ↑	Assay	Exposure time	Tested concentration [mmol/l]	Effective concentration [mmol/l]	IC50/EC50 [mmol/l]	Level of significance (*P*-value)	Number of exp.	Authors
**=**	trypan blue exclusion, light microscopy	15min	0.01 - 0.5	−	−	−	4	Sasagawa et al., 1984 (74).
**=**	trypan blue exclusion, light microscopy	15min	0.005 - 0.5	−	−	−	4	Sasagawa et al., 1985 (72)
**=**	trypan blue exclusion, light microscopy	30/120min	0.0005 - 1.5	−	−	−	2	Seow et al., 1994 (64).
**=**	lactate turnover, LDH release, UV spectrophotometric	15min	0.005 - 0.5	−	−	−	4	Sasagawa et al., 1985 (72)
**=**	lactate turnover, LDH release, UV spectrophotometric	15min	0.01 - 0.5	−	−	−	4	Sasagawa et al., 1984 (74)
**=**	MTT turnover, spectrophotometric	120min	0.0005 - 1.5	−	−	−	2	Seow et al., 1994 (64)
**PMA-stimulated =**	MTT turnover, spectrophotometric	120min	0.0005 - 1.5	−	−	−	2	Seow et al., 1994 (64)
**L220-stimulated =**	MTT turnover, spectrophotometric	4h	0.1	–	−	−	n.s.	Villalobos-Gomez et al., 2018 (48)
**(↑)**	pyruvate turnover, LDH release, UV Spectrophotometic	2h	10	(10)	−	n.d.	3 - 6	Bridges et al., 1977 (76)
**(↑)**	trypan blue exclusion, light microscopy	2h	10	(10)	−	n.d.	3 - 6	Bridges et al., 1977 (76)
**(↑)**	trypan blue exclusion, light microscopy	10min	0.000031 - 15	(3.1 - 15)	−	NS	3	Totti et al., 1984 (73)
**(↑)**	trypan blue exclusion, light microscopy	90min	0.0001 - 10	(1 - 10)	−	n.d.	5	Nowak et al., 1990 (68)
**↑**	trypan blue exclusion, light microscopy	45min	0.1 - 10	10	−	< 0.05	n.s.	Ryder et al., 1994 (65)
**(↑)**	MTT turnover, spectrophotometric	4h	0.1	(0.1)	−	n.d.	n.s.	Villalobos-Gomez et al., 2018 (48)
**↑**	released GAPDH, Western blot	4h	3.3	3.3	−	≤ 0.001	12	Gosh et al., 2019 (13)
**PG/VG-stimulated ↑**	released GAPDH, Western blot	4h	3.3	3.3	−	≤ 0.001	12	Gosh et al., 2019 (13)
**↓**	annexin V and PI stain, flow cytometry	4/24/36/54h	0.001	36/54h: 0.001	−	< 0.001 ✧	3	Xu et al., 2013 (51)
**↓**	trypan blue exclusion, light microscopy	24/48/72h	0.000001 - 0.01	24h: 0.0001/0.001 48h: 0.000001 - 0.01	−	24h: < 0.01 48h: < 0.01 ✧	6	Aoshiba et al., 1996 (62)
**↓**	trypan blue exclusion, light microscopy	24/48/72h	0.000001 - 0.01	24h: 0.0001/0.001 48h: 0.0001 - 0.01 72h: 0.001/0.01	−	24h: < 0.01 48h: < 0.01 72h: < 0.05 ✧	6	Aoshiba et al., 1996 (62)

= no effect; ↓ effect on neutrophils inhibited by nicotine; ↑ effect on neutrophils strengthened by nicotine. ✧ In case of kinetic studies, only the time points showing effects are shown. n.d., not determined; n.s., not stated.

**Table 3:** Influence of nicotine on apoptosis.

Effect = ↓ ↑	Assay	Exposure time	Tested concentration [mmol/l]	Effective concentration [mmol/l]	IC50/EC50 [mmol/l]	Level of significance (*P*-value)	Number of exp.	Authors
**=**	DNA gel electrophoresis	8h	0.5 - 22	−	−	−	n.s.	Yoshida et al., 1998 (59)
**↓**	acridine orange, ethidium bromide stain, fluorescence microscopy	24/48/72h	0.001	24h: 0.001	−	24h: < 0.05 ✧	6	Aoshiba et al., 1996 (62)
**↓**	electron microscopy	24/48/72h	0.001	24h: 0.001	−	24h: < 0.05 ✧	6	Aoshiba et al., 1996 (62)
**(↓)**	DNA gel electrophoresis	24h	0.001	(0.001)	−	−	1 repr.	Aoshiba et al., 1996 (62)
**↑**	DAPI stain, fluorescence microscopy	30min	0.6 – 18.5▵	18.5	−	7h: < 0.05 ✧	3	Mariggio et al., 2001 (58)
**(↑)**	DNA gel electrophoresis	30min	0.6 – 18.5▵	−	−	−	1 repr.	Mariggio et al., 2001 (58)
Fas ↓*	mRNA, RT-qPCR	12h	0.001	0.001	−	0.001	3	Tyagi et al., 2021 (46)
**Fas (↑)**	ELISA	30min	0.6 – 18.5▵	(18.5)	−	n.d.	3	Mariggio et al., 2001 (58)

= no effect; ↓ effect on neutrophils inhibited by nicotine; ↑ effect on neutrophils strengthened by nicotine. Specified subpopulation of *non adherent neutrophils in culture. ✧ In case of kinetic studies, only the time points with effects are shown. ▵ Nicotine concentration converted into molar values based on the molar mass of nicotine of 162.23 g/mol. n.d., not determined; n.s. not stated; repr., representative result shown.

**Table 4:** Influence of nicotine on chemotaxis.

Effect = ↓ ↑	Assay	Exposure time	Tested concentration [mmol/l]	Effective concentration [mmol/l]	IC50/EC50 [mmol/l]	Level of significance (*P*-value)	Number of exp.	Authors
**towards fMLP =**	traversing agarose	2h during movement	0.0001 - 0.1	−	–	n.d.	2 - 4	Gala et al., 1984 (75)
**towards fMLP ↑**	traversing agarose	90min preincubation	0.0001 - 0.01	0.0001 - 0.01	−	0.0001 mmol/l: < 0.05 0.001 - 0.1mmol/l: < 0.01	5	Nowak et al., 1990 (68)
**towards fMLP ↓**	traversing agarose	90min preincubation	0.1 - 10	10	−	< 0.01	5	Nowak et al., 1990 (68)
**towards fMLP ↑**	microchemotaxis chambers	24h preincubation	0.001	0.001	−	< 0.01	5	Aoshiba et al., 1996 (62)
**towards fMLP ↑**	modified Boyden Chamber	n.s.	0.00003 - 0.0308	0.00031 - 0.0308	−	< 0.001	15	Totti et al., 1984 (73)
**towards fMLP ↑**	modified Boyden Chamber	n.s.	0.0308	0.0308	−	< 0.01	15	Totti et al., 1984 (73)
**towards fMLP =**	modified Boyden Chamber	45min during movement	0.005 - 0.5	−	−	−	5	Sasagawa et al., 1985 (72)
**towards fMLP ↓**	traversing agarose	15min preincubation + 2h during movement	0.005 - 1.5	1 - 1.5	−	< 0.001	3	Seow et al., 1994 (64)
**towards C5fr ↑**	modified Boyden Chamber	n.s.	0.00003 - 0.0308	0.00031 - 0.0308	−	< 0.001	15	Totti et al., 1984 (73)
**towards ZAS ↑**	traversing agarose	90min preincubation	0.0001 - 0.01	0.0001 - 0.01	−	0.0001 mmol/l: < 0.05 0.001 mmol/l: < 0.01 0.01 mmol/l: < 0.05	5	Nowak et al., 1990 (68)
**towards ZAS ↓**	traversing agarose	90min preincubation	0.1 - 10	1 - 10	−	< 0.01	5	Nowak et al., 1990 (68)
**toward LTB4 ↓**	traversing agarose	15min preincubation + 2h during movement	0.005 - 1.5	1 - 1.5	−	< 0.001	3	Seow et al., 1994 (64)
**towards CXCL8 ↓**	traversing agarose	15min preincubation + 2h during movement	0.005 - 1.5	1 - 1.5	−	< 0.001	3	Seow et al., 1994 (64)
**toward CaAS ↓**	traversing agarose	15min preincubation + 2h during movement	0.005 - 1.5	1 - 1.5	−	< 0.001	3	Seow et al., 1994 (64)
**towards EAS ↓**	modified Boyden Chamber	2h during movement	2 - 10	6 - 10	IC50 = 3.5	< 0.05	3 - 6	Bridges et al., 1977 (76)
**towards nicotine (↑)**	traversing agarose	90min	0.0001 - 10	(0.0001-10)	−	n.d.	9	Nowak et al., 1990 (68)

= no effect; ↓ effect on neutrophils inhibited by nicotine; ↑ effect on neutrophils strengthened by nicotine. n.d., not determined; n.s., not stated.

**Table 5:** Influence of nicotine on general motility.

Effect = ↓ ↑	Assay	Exposure time	Tested concentration [mmol/l]	Effective concentration [mmol/l]	IC50/EC50 [mmol/l]	Level of significance (*P*-value)	Number of exp.	Authors
**Undirected movement (↓)**	traversing agarose	90min preincubation	0.0001 - 10	(0.01 – 10)	−	n.d.	n.s.	Nowak et al., 1990 (68)
**Undirected movement ↑**	trans-well system	2h during movement	0.6	0.6	−	< 0.01	3	Iho et al., 2003 (56)
**Undirected movement (↑)**	modified Boyden Chamber	n.s.	0.000031 - 10	(0.031 - 1)	−	n.d.	15	Totti et al., 1984 (73)
**Undirected movement =**	modified Boyden Chamber	45min during movement	0.005 - 0.5	−	−	–	5	Sasagawa et al., 1985 (72)
**Filtration time =**	cell filterability assay	10min	0.001	−	−	−	n.s.	Aoshiba et al., 1994 (66)
**Filtration time,** **O_2_-stimulated =**	cell filterability assay	10min	0.001	−	−	−	5	Aoshiba et al., 1994 (66)
**Filtration time,** **fMLP stimulated =**	cell filterability assay	2min	0.001	−	−	−	5	Aoshiba et al., 1994 (66).
**Filtration time,** **chloramineT-induced ↓**	cell filterability assay	10min	0.001	0.001	−	< 0.05	5	Aoshiba et al., 1994 (66).
**Filtration time,** **H_2_O_2_-induced ↓**	cell filterability assay	10min	0.001	0.001	−	< 0.05	5	Aoshiba et al., 1994 (66)
**Actin filament ↑**	flow cytometry	30sec, 30min	0.1 - 10	1-10	−	< 0.05, < 0.01	3	Ryder et al., 1994 (65)
**Actin filament,** **FLPEP-stimulated ↑**	flow cytometry	30sec	10	10	−	< 0.05	3	Ryder et al., 1994 (65)

= no effect; ↓ effect on neutrophils inhibited by nicotine; ↑ effect on neutrophils strengthened by nicotine. n.d., not determined; n.s., not stated.

**Table 6:** Influence of nicotine on adhesion molecule expression.

Effect = ↓ ↑	Assay	Exposure time	Tested concentration [mmol/l]	Effective concentration [mmol/l]	IC50/EC50 [mmol/l]	Level of significance (*P*-value)	Number of exp.	Authors
**CD62L =**	flow cytometry	15min	0.001 - 1	−	−	−	3	Ryder et al., 1998 (60)
**CD62L (↓)**	flow cytometry	15min	0.001 - 0.01	(0.001 - 0.01)	−	n.d.	1	Speer et al., 2002 (57)
**CD62L, C5a-stimulated ↓**	flow cytometry	20min	0.001	0.001	−	< 0.05	7	Vukelic et al., 2013 (19)
**CD18 =**	flow cytometry	15min	0.001 - 1	−	−	−	4	Ryder et al., 1998 (60)
**CD11a (↓)**	flow cytometry	15min	0.001 - 0.01	(0.001 - 0.01)	−	n.d.	1	Speer et al., 2002 (57)
**CD11b (↓)**	flow cytometry	15min	0.001 - 0.01	(0.001 - 0.01)	−	n.d.	1	Speer et al., 2002 (57)
**CD11b, C5a-stimulated ↓**	flow cytometry	20min	0.001	0.001	−	< 0.05	7	Vukelic et al., 2013 (19)

= no effect; ↓ effect on neutrophils inhibited by nicotine; ↑ effect on neutrophils strengthened by nicotine. n.d., not determined; n.s., not stated.

**Table 7:** Influence of nicotine on eicosanoid synthesis.

Effect = ↓ ↑	Assay	Exposure time	Tested concentration [mmol/l]	Effective concentration [mmol/l]	IC50/EC50 [mmol/l]	Level of significance (*P*-value)	Number of exp.	Authors
**LTB4, A23187-stimulated ↓**	secreted, HPLC	15min	0.0000005 - 0.5	0.0000005 - 0.5	IC50 = 0.0321	< 0.05	6	Saareks et al., 1993 (67)
**LTB4, A23187-stimulated ↑**	secreted, enzyme immunoassay	15min	0.0005 - 1.5	1 - 1.5	−	< 0.001	3	Seow et al., 1994 (64)
**LTB4, A23187-stimulated ↑**	secreted, isotope-labelled GC-MS	1/3/8/12h	0.000062▵	0.000062	−	< 0.05	5	Loke et al., 2012 (53)
**LTB4, cotinine/A23187-stimulated (↑)**	secreted, isotope-labelled GC-MS	1/3/8/12h	0.000062▵	(0.000062)	−	n.d.	5	Loke et al., 2012 (53)
**PGE2, A23187-stimulated ↑**	secreted, radio immunoassay	15min	0.0000005 - 0.5	0.0000005 - 0.5	IC50 = 0.0365	< 0.05	6	Saareks et al., 1993 (67)
**PGE2, concanavalin A-stimulated ↑**	secreted, enzyme immunoassay	15min	0.0005 - 1.5	0.5 - 1.5	−	0.5 mmol/l: < 0.01 1 - 1.5 mmol/l: < 0.001	3	Seow et al., 1994 (64)

= no effect; ↓ effect on neutrophils inhibited by nicotine; ↑ effect on neutrophils strengthened by nicotine. ▵ Nicotine concentration converted in to molar values based on the molar mass of nicotine of 162.23 g/mol. n.d., not determined.

**Table 8:** Influence of nicotine on cytokine/chemokine expression.

Effect = ↓ ↑	Assay	Exposure time	Tested concentration [mmol/l]	Effective concentration [mmol/l]	IC50/EC50 [mmol/l]	Level of significance (*P*-value)	Number of exp.	Authors
**CXCL8 ↑**	secreted protein, ELISA	16h	0.01 - 3	0.1 - 3	EC50 = 1.89	0.1mmol/l: < 0.05 0.5 - 3mmol/l: < 0.01	6	Iho et al., 2003 (56)
**CXCL8 ↑ ✧**	secreted protein, ELISA	1/2/3/4/16/22h	3	3/4/16/22h: 3	−	< 0.01	3	Iho et al., 2003 (56)
**CXCL8 ↑**	secreted protein, ELISA	16h	2.5	2.5	−	< 0.01	3	Iho et al., 2003 (56)
**CXCL8 ↑**	mRNA, RT-qPCR	2h	1 - 2	−	−	−	1 repr.	Iho et al., 2003 (56)
**IFN-γ=**	secreted protein, ELISA	4h	0.1	−	−	−	3	Villalobos-Gomez et al., 2018 (48)
**IFN-γ, L220-induced (↓)**	secreted protein, ELISA	4h	0.1	(0.1)	−	NS	3	Villalobos-Gomez et al., 2018 (48)
**IL-1ß =**	secreted protein, ELISA	4h	0.1	−	−	−	3	Villalobos-Gomez et al., 2018 (48)
**IL-1ß, L220-induced ↓**	secreted protein, ELISA	4h	0.1	0.1	−	≤ 0.05	3	Villalobos-Gomez et al., 2018 (48)
**TNF-α =**	secreted protein, ELISA	4h	0.1	−	−	−	3	Villalobos-Gomez et al., 2018 (48)
**TNF-α, L220-induced =**	secreted protein, ELISA	4h	0.1	−	−	−	3	Villalobos-Gomez et al., 2018 (48)
**IL-10 =**	secreted protein, ELISA	4h	0.1	−	−	−	3	Villalobos-Gomez et al., 2018 (48)
**IL-10, L220-induced =**	secreted protein, ELISA	4h	0.1	−	−	−	3	Villalobos-Gomez et al., 2018 (48)
**LCN2 ↑**	secreted protein, ELISA	24h	0.001	0.001	−	0.003	3	Tyagi et al., 2021 (46)
CCL2 ↑**	mRNA, RT-qPCR	12h	0.001	0.001	−	0.03	3	Tyagi et al., 2021 (46)
CCL3 ↓*	mRNA, RT-qPCR	12h	0.001	0.001	−	0.001	3	Tyagi et al., 2021 (46)

= no effect; ↓ effect on neutrophils inhibited by nicotine; ↑ effect on neutrophils strengthened by nicotine. Specified subpopulation of *non adherent or **adherent neutrophils in culture. ✧ In case of kinetic studies, only the time points showing effects are shown. NS, not significant; repr., representative result shown.

**Table 9:** Influence of nicotine on NET formation.

Effect = ↓ ↑	Assay	Exposure time	Tested concentration [mmol/l]	Effective concentration [mmol/l]	IC50/EC50 [mmol/l]	Level of significance (*P*-value)	Number of exp.	Authors
**=**	SYTOX green DNA stain, spectrophotometric	30min	0.0062 - 0.062▵	−	−	−	5	White et al., 2018 (47)
**=**	SYTOX green DNA stain, fluorescence microscopy	30min	0.0062 - 0.062▵	−	−	−	1 repr.	White et al., 2018 (47)
**PMA-stimulated =**	SYTOX green DNA stain, spectrophotometric	30min	0.0062 - 0.062▵	−	−	−	5	White et al., 2018 (47)
**PMA-stimulated =**	SYTOX green DNA stain, fluorescence microscopy	30min	0.0062 - 0.062▵	−	−	−	1 repr.	White et al., 2018 (47)
**HOCl-stimulated =**	SYTOX green DNA stain, spectrophotometric	30min	0.0062 - 0.062▵	−	−	−	5	White et al., 2018 (47).
**HOCl-stimulated =**	SYTOX green DNA stain, fluorescence microscopy	30min	0.0062 - 0.062▵	−	−	−	1 repr.	White et al., 2018 (47).
**↑**	DAPI staind area, fluorescence microscopy	10h	0.01 - 1	0.01 - 1	−	< 0.001	6	Hosseinzadeh et al., 2016 (50)
**↑**	SYTOX green DNA stain, spectrophotometric	1 -10h	0.01	5- 10h: 0.01	−	< 0.05 ✧	6	Hosseinzadeh et al., 2016 (50)
**↑**	SYTOX green DNA stain, spectrophotometric	10h	0.001 - 2	0.01 - 2	−	0.01/0.1 mmol/l: < 0.05 1 mmol/l: < 0.01 2 mmol/l: < 0.001	6	Hosseinzadeh et al., 2016 (50)
**↑**	SYTOX green DNA stain, spectrophotometric	4h	1- 20	4 - 20	−	vs 1 mmol/l: < 0.0001	≥ 2	Lee et al., 2017 (49)
**↑**	MPO-DNA complexes released, ELISA	4h	1 - 5	3 - 5	−	< 0.0001	≥ 2	Lee et al., 2017 (49)
**(↑)**	Real-time fluorescence video microscopy	1-3h	2.5	2.5 ✧	−	−	≥ 2	Lee et al., 2017 (49)
**TNF-α -stimulated ↑**	SYTOX green DNA stain, spectrophotometric	4h	1	1	−	< 0.005	≥ 2	Lee et al., 2017 (49)
**immune complex-stimulated ↑**	SYTOX green DNA stain, spectrophotometric	4h	1	1	−	< 0.001	≥ 2	Lee et al. (49),.
**↑**	SYTOX green DNA stain, fluorescence microscopy	5-6h (every 30 min)	0.00062 - 0.0018▵	0.0012 - 0.0018▵	−	< 0.05/0.01	≥ 5	Aspera-Werz et al., 2022 (45)
**↑**	SYTOX green DNA stain, MPO stained localization, fluorescence microscopy	5h	0.00062 - 0.0018▵	−	−	−	1 repr.	Aspera-Werz et al., 2022 (45)

= no effect; ↓ effect on neutrophils inhibited by nicotine; ↑ effect on neutrophils strengthened by nicotine. ▵ Nicotine concentration converted into molar values based on the molar mass of nicotine of 162.23 g/mol. ✧ In case of kinetic studies, only the time points showing effects are shown. repr., representative result shown.

**Table 10:** Influence of nicotine on phagocytosis.

Effect = ↓ ↑	Assay	Exposure time	Tested concentration [mmol/l]	Effective concentration [mmol/l]	IC50/EC50 [mmol/l]	Level of significance (*P*-value)	Number of exp.	Authors
**Uptake bacteria =**	uptake of 3H-thymididine-labeled F. nucleatum	10/20/30/60min	6.2 ✦	−	−	−	2	Pabst et al., 1995 (63)
**Uptake of microsphere ↓**	flow cytometry	60min	0.1 - 1	1	−	< 0.001	4	Ryder et al., 1994 (65)
**Uptake bacteria ↓**	phase-contrast microscopy	15min	0.005 - 1.5	0.5 - 1.5	−	< 0.001	3	Seow et al., 1994 (64)
**Uptake IgG-SRBC, C5a-stimulated ↓**	flow cytometry	20min	0.00001 - 0.001	0.001	−	< 0.05	3-6	Vukelic et al., 2013 (19)

= no effect; ↓ effect on neutrophils inhibited by nicotine; ↑ effect on neutrophils strengthened by nicotine. ✦ Nicotine concentrations declared by the author.

**Table 11:** Influence of nicotine on ROS generation.

Effect = ↓ ↑	Assay	Exposure time	Tested concentration [mmol/l]	Effective concentration [mmol/l]	IC50/EC50 [mmol/l]	Level of significance (*P*-value)	Number of exp.	Authors
**=**	luminol turnover, chemilumimetric	3h	0.01 - 1	−	−	−	n.d.	Hosseinzadeh et al., 2016 (50)
**=**	luminol turnover, chemilumimetric	30min	0.0062 – 0.062▵	−	−	−	6	Matthews et al., 2011 (54)
**=**	isoluminol turnover, chemilumimetric	30min	0.0062 – 0.062▵	−	−	−	6	Matthews et al., 2011 (54)
**↓**	DCFH turnover, spectrophotometric	20min	0.0001 - 0.0003▵	0.0002 - 0.0003	−	0.0002 mmol/l: < 0.05 0.0003 mmol/l: < 0.01	6	Aspera-Werz et al., 2022 (45)
**=**	luminol turnover, chemilumimetric	60min	0.49▵	−	−	NS	3	Al-Shibani et al., 2011 (55)
***P. gingivalis*-stimulated =**	luminol turnover, chemilumimetric	60min	0.49▵	−	−	−	3	Al-Shibani et al., 2011 (55)
***F. nucleatum*-stimulated =**	luminol turnover, chemilumimetric	150min	0.0062 – 0.062▵	−	−	−	6	Matthews et al., 2011 (54)
***F. nucleatum*-stimulated =**	isoluminol turnover, chemilumimetric	150min	0.0062 – 0.062▵	−	−	−	6	Matthews et al., 2011 (54)
**OZA-stimulated ↓**	luminol turnover, chemilumimetric	10min	0.01 - 0.5	0.05 - 0.5	−	0.05 mmol/l: < 0.05 0.1 - 0.5 mmol/l: < 0.01	4	Sasagawa et al., 1984 (74)
**fMLP/CB-stimulated ↓**	chemilumimetric	5min	1 - 10	10	IC50 = 3	< 0.001	5	Srivastava et al., 1989 (70)
**O_2_^-^ =**	lucigemin turnover, chemilumimetric	30min	0.0062 - 0.062▵	−	−	−	5	Matthews et al., 2012 (52)
**O_2_^-^ ↓**	cytochrome C turnover, spectrophotometric	10min	0.000031 - 15	3.1 - 15	−	< 0.01	3	Totti et al., 1984 (73)
**O_2_^-^ ↑**	cytochrome C turnover, spectrophotometric	5-90min	3	3	−	< 0.01	3	Iho et al., 2003 (56)
**O_2_^-^, ops S. aureus-stimulated =**	lucigemin turnover, chemilumimetric	150min	0.0062 - 0.062▵	−	−	−	4	Matthews et al., 2012 (52)
**O_2_^-^, F. nucleatum-stimulated =**	lucigemin turnover, chemilumimetric	150min	0.0062 - 0.062▵	−	−	−	4	Matthews et al., 2012 (52)
**O_2_^-^, LPS-stimulated =**	lucigemin turnover, chemilumimetric	150min	0.0062 - 0.062▵	−	−	−	4	Matthews et al., 2012 (52)
**O_2_^-^, PMA-stimulated =**	ferricytochrome C turnover, spectrophotometric	n.s.	0.0005 - 1.5	−	−	−	3	Seow et al., 1994 (64)
**O_2_^-^, PMA-stimulated ↑**	cytochrome C turnover, spectrophotometric	10min	0.00001 - 0.01	0.00001 - 0.01	−	< 0.05	5	Jay et al., 1986 (71)
**O_2_^-^, fMLP-stimulated ↑**	cytochrome C turnover, spectrophotometric	10min	0.00001 - 0.01	0.00001 - 0.01	−	< 0.05	5	Jay et al., 1986 (71)
**O_2_^-^, fMLP-stimulated ↓**	cytochrome C turnover, spectrophotometric	10min	0.000031 - 15	3.1 - 15	−	< 0.01	3	Totti et al., 1984 (73)
**O_2_^-^, fMLP-stimulated/CB ↓**	ferricytochrome C turnover, spectrophotometric	2min	0.005 - 0.5	0.05 - 0.5	−	0.05 mmol/l: < 0.05 0.1 - 0.5 mmol/l: < 0.01	5	Sasagawa et al., 1985 (72)
**O_2_^-^, fMLP-stimulated/CB (↓)**	ferricytochrome C turnover, spectrophotometric	2min	0.1	(0.1)	−	n.d.	4	Sasagawa et al., 1985 (72)
**O_2_^-^, fMLP stimulated ↑**	cytochrome C turnover, spectrophotometric	24.5h	0.001	0.001	−	< 0.05	3	Aoshiba et al., 1996 (62)
**O_2_^-^, LPS/PMA-stimulated ↓**	cytochrome C turnover, spectrophotometric	7min	0.62 - 6.2 ✦	0.62 - 6.2	−	< 0.001	2	Pabst et al., 1995 (63)
**O_2_^2-^, PMA-stimulated ↓**	phenol red turnover, spectrophotometric	1h	1.86 - 6.2 ✦	1.86 - 6.2	−	< 0.05	2	Pabst et al., 1995 (63)
**H_2_O_2_ ↑**	amplex red turnover, spectrophotometric	25min - 15h	2.5	2.5	−	increase of > 1.2-fold	2	Iho et al., 2003 (56).
**↑**	DHR123 stain, flow cytometry	25min - 12h	2.5	2.5	−	increase of > 1.2-fold	2	Iho et al., 2003 (56)
**C5a-stimulated ↓**	DHR123 stain, flow cytometry	5min	0.00001 - 1	0.001 - 1	−	0.001 mmol/l: < 0.001 0.01 - 1 mmol/l: < 0.05	5	Vukelic et al., 2013 (19)
**Fcy-receptor cross-linked ↓**	DHR123 stain, flow cytometry	5min	0.001	0.001	−	< 0.05	5	Vukelic et al., 2013 (19)

= no effect; ↓ effect on neutrophils inhibited by nicotine; ↑ effect on neutrophils strengthened by nicotine. ✦ Nicotine concentrations declared by the author. ▵ Nicotine concentration converted in to molar values based on the molar mass of nicotine of 162.23 g/mol. n.d., not determined; NS, not significant.

**Table 12:** Influence of nicotine on antimicrobial activity.

Effect = ↓ ↑	Assay	Exposure time	Tested concentration [mmol/l]	Effective concentration [mmol/l]	IC50/EC50 [mmol/l]	Level of significance (*P*-value)	Number of exp.	Authors
***A. actiomycetemcomitans* ↓**	(aerobic) bacteria colonies formed from lysed cells	30min	0.62 - 6.2 ✦	0.62 - 6.2	−	< 0.002	2	Pabst et al., 1995 (63)
***F. nucleatum* ↓**	(aerobic) bacteria colonies formed from lysed cells	30min	0.62 - 6.2 ✦	0.62 - 6.2	−	< 0.002	2	Pabst et al., 1995 (63)
***A. naeslundii* ↓**	(aerobic) bacteria colonies formed from lysed cells	30min	0.62 - 6.2 ✦	0.62 - 6.2	−	< 0.002	2	Pabst et al., 1995 (63)
***F. nucleatum* =**	(anaerobic) bacteria colonies formed from lysed cells	30min	6.2 ✦	−	−	−	2	Pabst et al., 1995 (63)
***A. naeslundii* =**	(anaerobic) bacteria colonies formed from lysed cells	30min	6.2 ✦	−	−	−	2	Pabst et al., 1995 (63)

= no effect; ↓ effect on neutrophils inhibited by nicotine; ↑ effect on neutrophils strengthened by nicotine. ✦ Nicotine concentrations declared by the author.

**Table 13:** Influence of nicotine on degrading enzyme release.

Effect = ↓ ↑	Assay	Exposure time	Tested concentration [mmol/l]	Effective concentration [mmol/l]	IC50/EC50 [mmol/l]	Level of significance (*P*-value)	Number of exp.	Authors
*β*-Glucuronidase								
**↓**	phenolphtalein glucuronide turnover, spectrophotometric	2h	10	(10)	−	n.d.	3 - 6	Bridges et al., 1977 (76)
**=**	MUG turnover, spectrophotometric	15min	0.005 - 0.5	−	−	−	5	Sasagawa et al. (72),
**↑**	phenolphtalein glucuronide turnover, spectrophotometric	60min	0.003 – 9.2 ▵	3.1 – 9.2	−	< 0.01	3	Seow et al., 1994 (64)
**fMLP/CB-stimulated ↓**	MUG turnover, spectrophotometric	15min	0.005 - 0.5	0.05 - 0.5	−	0.05 mmol/l: < 0.05 0.1 - 0.5 mmol/l: < 0.01	5	Sasagawa et al., 1985 (72)
Lysozyme								
**↑**	n.s., spectrophotometric	60min	0.003 – 9.2 ▵	3.1 – 9.2	−	< 0.01	3	Seow et al., 1994 (64)
**CB-stimulated =**	n.s., spectrophotometric	10min	0.000031 - 15	(3.1 - 15)	−	NS	3	Totti et al., 1984 (73)
**fMLP/CB-stimulated ↓**	n.s., spectrophotometric	10min	0.000031 - 15	(15)	−	< 0.01	3	Totti et al., 1984 (73)
Myeloperoxidase								
**=**	TMB turnover, spectrophotometric	15min	0.005 - 0.5	−	−	−	5	Sasagawa et al., 1985 (72)
**CB-stimulated =**	n.s., spectrophotometric	10min	0.000031 - 15	−	−	−	3	Totti et al., 1984 (73)
**fMLP/CB-stimulated ↓**	n.s., spectrophotometric	10min	0.000031 - 15	(3.1 - 15)	−	< 0.01	3	Totti et al., 1984 (73)
**fMLP/CB-stimulated ↓**	TMB turnover, spectrophotometric	15min	0.005 - 0.5	0.1 - 0.5	−	< 0.01	5	Sasagawa et al. (72),
**A23187-stimulated # ↑**	tyrosine metabolite, isotope-labelled GC-MS	0.5 -7h	0.000062▵	0.000062	−	< 0.05	5	Loke et al., 2012 (53)
**Cotinine/A23187-stimulated # (↑)**	tyrosine metabolite, isotope-labelled GC-MS	0.5 -7h	0.000062▵	(0.000062)	−	n.d.	5	Loke et al., 2012 (53)
Elastase								
**↑**	fluorogenic substate turnover, n.s.	2min	−	−	EC50 = 3.9	−	9	Gosh et al., 2019 (13)
**↑**	fluorogenic substate turnover, n.s.	4h	3.3	3.3	−	≤ 0.05	12	Gosh et al., 2019 (13)
**↑**	Western blot	4h	3.3	3.3	−	≤ 0.05	12	Gosh et al., 2019 (13)
**↑**	enzyme-immunoassay, spectrophotometric	60min	0.003 – 9.2 ▵	3.1 – 9.2	−	< 0.01	3	Seow et al., 1994 (64)
**(↑)**	n.s., spectrophotometric	90min	0.308 - 6.2	(3.08 - 6.2)	−	−	10	Murphy et al., 1998 (61)
**CB-stimulated =**	elastin turnover, spectrophotometric	10min	0.000031 - 15	−	−	−	3	Totti et al., 1984 (73)
**fMLP/CB-stimulated =**	elastin turnover, spectrophotometric	10min	0.000031 - 15	–	−	–	3	Totti et al., 1984 (73)
**PMA-stimulated ↑**	elastin turnover, spectrophotometric	5min	0.0004 - 4	0.4 - 4	−	< 0.001	4	Nowak et al., 1990 (69)
**PG/VG-stimulated ↑**	fluorogenic substate turnover, n.s.	4h	3.3	3.3	−	≤ 0.05	12	Gosh et al., 2019 (13)
**PG/VG-stimulated ↑**	Western blot	4h	3.3	3.3	−	≤ 0.05	12	Gosh et al., 2019 (13).

= no effect; ↓ effect on neutrophils inhibited by nicotine; ↑ effect on neutrophils strengthened by nicotine. The effects are shown in parentheses in case they appeared to be biologically relevant despite the lack statistics of a statistic proof. n.s., not stated; n.d., not determined; NS, not significant.

### 3.1 General cell death/damage

General cell death/damage of nicotine-exposed neutrophils has been studied in eleven research articles (13, 48, 51, 62, 64, 65, 68, 72–74, 76). The results are displayed in **Table 2**. At the first glance, the results appear contradictory, but closer inspection revealed that the influence on general cell death/damage was dependent on the nicotine concentration and exposure time. Six articles revealed that high concentrations (>3 mmol/l) of nicotine, already when given for a very short time (down to 10 min), lead to increased cell death/damage of neutrophils (13, 48, 65, 68, 73, 76). Four research articles demonstrated that exposure to nicotine at concentrations of 0.0005 – 1.5 mmol/l for 15 – 120 min didn´t interfere with general cell death/damage (48, 64, 72, 74). Interestingly, two research articles showed that low nicotine concentrations (shown for 0.000001 - 0.01 mmol/l) given for long exposure times (≥ 24h) led to reduced general cell death/damage, i.e., supported the neutrophil survival (51, 62). Aoshiba et al. and Xu et al. raised the hypothesis that activation of non-cholinergic receptor and prevention of Akt dephosphorylation are involved in the reduced cell death of neutrophils exposed to low doses of nicotine (51, 62).

### 3.2 Apoptosis

As described above, neutrophils have a limited physiological life expectancy. Apoptosis is a finely regulated programmed cell death that does not lead to inflammation or damage of neighboring cells. Thus, alteration of this process can result in inflammation and impaired tissue homeostasis (77). Three research articles investigated the effect of nicotine on neutrophil apoptosis and the results are summarized in **Table 3**. Aoshiba et al. found that the reduction of general cell death/damage due to long-term exposure to low nicotine concentrations goes along with a reduced apoptosis rate as assessed using (i) acridine orange/ethidium bromide staining, (ii) electron microscopy showing chromatin aggregation, nuclear prominence and cytoplasmic vacuolation, and (iii) DNA fragmentation (62). Yoshida et al. suggested a lacking impact of even very high concentrations of nicotine on apoptosis; however, this was based on a non-indicated number of performed experiments and a low-quality visualization of DNA fragmentation (59). At cell-toxic nicotine concentrations, Mariggio et al. reported an increase in neutrophil apoptosis, evident by increased DNA fragmentation (58). In line, the authors detected an increased Fas protein expression in the exposed neutrophils (58). Tyagi et al. reported a decrease in Fas mRNA expression in adherent neutrophils after an extended low-dose nicotine exposure (12 h) (46). Fas is a cell death receptor that induces apoptosis after binding of Fas ligand (78).

As the above described results indicate, concentrations >3 mmol/l significantly affect cell viability and are considered to be cytotoxic. Therefore, the effects of nicotine in concentrations below 3 mmol/l were discussed in the subsequent part of the manuscript.

### 3.3 Chemotaxis

Under physiological conditions, neutrophils circulate in the blood. When tissue damage or inflammation occurs locally, specific mediators are released that attract neutrophils to the site of damage/inflammation in a process so-called chemotaxis (79). These chemotactic mediators can be produced by different cells (e.g. mast cells, macrophages, fibroblasts) or arise in defined processes (e.g., release from damaged cells, complement activation) and have different chemical properties. We identified eight research articles dealing with the influence of nicotine on chemotaxis (56, 62, 64, 68, 72, 73, 75, 76). The results are displayed in **Table 4**. While high nicotine concentrations reduced the movement of the cells towards chemoattractants such as n-formylmethionyl-leucyl-phenylalanine (fMLP) and complement products (isolated complement factor 5 fragments or fragments generated in activated serum (ZAS)), concentrations ≤ 0.031 mmol/l mostly had an increasing effect (62, 68, 73). Notably, nicotine itself was suggested to act as a chemoattractant for neutrophils, as the movement of neutrophils towards 0.6 mmol/l nicotine was enhanced in the study by Nowak et al. (68). Nicotine studies, in which no gradient of chemoattractant was methodically built up, are dealt with in the chapter ‘General motility’.

### 3.4 General motility

Neutrophils patrol the blood and are able to enter the tissue rapidly upon a signal. For the movement through the endothelia, neutrophils form pseudopods, in which actin contributes to the change in shape (80). Six research articles investigated aspects of general motile function of neutrophils (56, 65, 66, 68, 72, 73). The results are displayed in **Table 5**. Aoshiba et al. studied the time of neutrophils to pass through micropore membrane (66). They revealed that nicotine itself at rather high concentrations had no influence. Furthermore, nicotine did not influence the delay in the filtration time induced by superoxide (O_2_^-^) and fMLP. In contrast, the increase in motility of neutrophils exposed to model oxidants such as chloramine T and hydrogene peroxide (H_2_O_2_) was found to be counteracted by nicotine in this study. Other studies investigating the spontaneous migration of neutrophils gave contrasting results (56, 68, 73). Ryder et al. studied the relative cellular content of filamentous actin (F-actin), the authors consider as an early indicator of the neutrophil movement. They found an increase in F-actin when the cells were exposed to nicotine and a synergistic action of nicotine with a hexapeptide agonist for the formyl peptide receptor in this respect (65).

Generally, the assessment of the directed (chemotaxis, see above) and undirected movement of neutrophils may be difficult during nicotine exposure if we assume that nicotine itself may be chemoattractive for these cells (68).

### 3.5 Adhesion molecule expression

Adhesion molecules play an essential role in the exit of neutrophils from blood vessels (diapedesis) and in their migration to the site of damage/inflammation in the tissue (81). Prominent neutrophil adhesion molecules come from the group of selectins (CD62L, P-selectin glycoprotein ligand 1) and integrins (as heterodimeric complexes, containing CD18 and either CD11a, CD11b or CD11c). Selectins on neutrophils bind to their ligands on endothelial cells and mediate initial attachment and subsequent rolling, which is followed by a more stable binding, mediated by integrins. CD11b/CD18 and CD11c/CD18 also enable neutrophils to migrate through the tissue and phagocytose bacteria opsonized with complement products. Three research articles examined the influence of nicotine on the expression of neutrophil’s adhesion molecules using flow cytometry (19, 57, 60) and the results are displayed in **Table 6**. Ryder et al. reported no regulation of CD62L, while Speer et al. and Vulkelic et al. found CD62L to be downregulated after nicotine exposure alone or in combination with C5a stimulation of the neutrophils. The same three articles investigated possible changes in the integrin expression and found no alteration of the CD18 expression. CD11a was downregulated at 0.001 mmol/l nicotine and completely lost when higher nicotine concentration (0.01 mmol/l) were used. CD11b expression was found to be progressively diminished following nicotine exposure either alone or with C5a as a co-stimulus (19, 57, 60).

### 3.6 Eicosanoid synthesis

Eicosanoids [e.g., leukotriene (LT), prostaglandin (PG)] are organic chemical compounds that are formed from polyunsaturated fatty acids (82). They can act in an autocrine and paracrine manner and influence various functions of target cells (e.g., chemotaxis of neutrophils). The search retrieved three publications in which the possible impact of nicotine on the eicosanoid synthesis of stimulated neutrophils was investigated (53, 64, 67). The results are displayed in **Table 7**. Seow et al., who used short-term exposure to higher concentrations, and Loke et al., who used lower concentrations and longer exposure times, reported that nicotine led to an increase in LTB4 synthesis in calcium ionophore A23187-stimulated neutrophils (53, 64). On the other hand, Saareks et al. reported a dose-dependent decrease in LTB4 synthesis in A23187-stimulated neutrophils (67). Two of these publications described that PGE2 production was increased by nicotine in calcium ionophore A23187- or concanavalin A-stimulated neutrophils (64, 67).

### 3.7 Cytokine/chemokine expression

Neutrophils can express a variety of pro-/anti-inflammatory cytokines and chemokines, including IL-1β, IL-10, tumor-necrosis factor (TNF)-α, lipocalin (LCN)-2, and BAFF, usually upon stimulation (41, 83, 84). We identified three research articles that investigated the potential effect of nicotine on neutrophil cytokine/chemokine expression (46, 48, 56). The results are displayed in **Table 8**. Iho et al. reported an elevated CXCL8 secretion by neutrophils following nicotine exposure, with an EC50 of 1.9 mmol/l (56). This would suggest that in addition to nicotine’s effect on chemotaxis towards chemoattractants present in the infected/inflamed tissue, this alkaloid also triggers the neutrophils to produce its own attractant. No influence was found on the secretion of interferon(IFN)-γ, IL-1β, tumor-necrosis factor (TNF)-α, and interleukin(IL)-10 in a study that used a single moderate concentration (0.1 mmol/l) of nicotine and a short exposure time (4h). In the same study, the secretion of IFN-γ and IL-1β induced by the 220kDa lectin of *Entamoeba histolytica* (L220) was even abrogated in the presence of nicotine (48). A decreased production of IL-1β, which acts on almost any cell type (85), would result in dampened non-specific inflammation. Applying extended exposure times (12-24h), Tyagi et al. found elevated STAT3-dependent release of LCN2 when the cells were exposed to only 0.001 mmol/l nicotine. LCN2, produced in large quantities in the skin of PPP and HS patients, is involved in the induction of inflammatory pain and supports the tissue infiltration of neutrophils, thereby mediating a positive feedback loop (40, 86). The authors further reported a nicotine-induced shift of non-adherent to adherent neutrophils, which was associated with a decreased CCL3 and an increased CCL2 expression respectively. A shift to CCL2 would result in the attraction of a broader spectrum of immune cells. CCR2, the receptor through which CCL2 acts, is expressed by numerous immune cell types, including monocytes, T cells, NK cells, basophils, mast cells and dendritic cells (87, 88).

### 3.8 NET formation

Neutrophils can release large, web-like structures called neutrophil extracellular traps (NETs) which consist of decompensated chromatin loaded with cytosolic and granular proteins and often let to the death of the cells (89). Four studies were found that deal with the effect of nicotine on the NET formation (45, 47, 49, 50). The results are displayed in **Table 9**. NET formation was detected by DNA stains (45, 47, 49, 50) and released DNA-MPO complexes (49). Three research articles demonstrated that nicotine on its own induces NETs (45, 49, 50) at a wide range of concentrations (as low as 0.01 mmol/l (50) or 0.0012 mmol/l (45) and up to cytotoxic concentrations (49)), using exposure times of 4 to 10 h. Lee et al. further demonstrated that TNF-α pretreatment and costimulation with immune complexes of citrullinated histone 2B increased the effect of nicotine on NET formation (49). The absence of a nicotine effect on constitutive and phorbol 12-myristate 13-acetate (PMA)- or hypochlorous acid (HOCl)-stimulated NET formation in neutrophils demonstrated by White et al. let us suggest that the exposure time of 30 min chosen in this study was too short (47). In fact, in a real-time fluorescence video, Lee et al. demonstrated that formation of NETs started at around 1h in nicotine-exposed neutrophils (49).

### 3.9 Phagocytosis

Neutrophils engulf prokaryotic and eukaryotic pathogens in a phagosome formed by the plasma membrane and then kill them through various mechanisms after granular vesicles fuse with the phagosome. The production of superoxides, which activate myeloperoxidase (MPO) to produce hypochlorite, and several effector proteins are involved in pathogen killing (90). Four articles described investigations into the influence of nicotine on the phagocytic activity of neutrophils, using microspheres, bacteria and immune globuline-opsonized sheep erythrocytes (IgG-opsonized-SRBC) (19, 63–65). Three of these articles reported an impaired phagocytic function when nicotine concentrations in the non-cytotoxic range (see above) were used (19, 64, 65). The results are displayed in **Table 10**. The reduced C5a-costimulated internalization of IgG-opsonized-SRBC suggests an inhibiting influence of nicotine on the Fc-γ receptor-mediated phagocytosis by neutrophils (19).

### 3.10 ROS formation

Neutrophils have the potential to generate a variety of ROS, including O_2_^-^ and H_2_O_2_. ROS are necessary for the killing of ingested microorganisms within the phagolysosome. However, their release into the tissue can induce tissue destruction (91). The literature search retrieved fifteen articles investigating nicotine-induced changes in the ROS release by neutrophils (19, 45, 50, 52, 54–56, 62–64, 70–74). The results are displayed in **Table 11**. Measurements were done extracellularly with and without the cells present. Time and duration of nicotine addition, as well as the assay for ROS measurement greatly varied. The results published are contradictory. No clear effect was seen, even when the studies were stratified by nicotine concentration, nicotine exposure time, costimulation or assessed ROS species.

### 3.11 Antimicrobial activity

Neutrophils have diverse mechanisms to exhibit antimicrobial properties. These include phagocytosis, ROS formation, cytokine release, exosome release and degranulation as well as NET formation (90). One single study examining changes in net antimicrobial activity of nicotine-exposed neutrophils was identified (63) and the results are summarized in **Table 12**. The authors assessed the bacteria colony formation of lysed neutrophils, which were previously exposed to *Actinobacillus actiomycetemcomitans*, *Fusobacterium nucleatum*, and *Actinomyces naeslundii* under anaerobic and aerobic conditions, to demonstrate that nicotine inhibited the neutrophil killing of the bacteria under aerobic conditions. We critically note the experimental setting, with either neutrophils that may be damaged under anaerobe conditions or obligate anaerobe bacteria like *Fusobacteri*a that may be damaged of under aerobe conditions, the low number of performed experiments in this study (63) and the lack of independent studies confirming these findings.

### 3.12 Degrading enzyme release

The arsenal of degrading enzymes found in different types of neutrophilic granules is crucial for the effectiveness of neutrophils against microorganisms. On the other hand, when released, these enzymes might induce tissue destruction. Nine research articles investigated the effect of nicotine on the release of enzymes from the neutrophils, including hydrolases, peroxidases, and proteases (13, 45, 53, 61, 64, 69, 72, 73, 76). The results are displayed in **Table 13**.

#### 3.12.1 β-Glucuronidase

β-glucuronidase splits glycosaminoglycans and is located in the primary granules of neutrophils. β-glucuronidase has been found to be released during bacterial infections (92, 93). The influence of non-cytotoxic concentrations of nicotine on the release of β-glucuronidase by neutrophils was investigated in two research articles. The results are contradictory as Seow et al. found an increased release (64), while Sasagawa et al. reported no influence on β-glucuronidase release (but an increase induced by cytotoxic nicotine concentrations) (72). Furthermore, Sasagawa et al. showed that nicotine reduced the β-glucuronidase release in neutrophils stimulated with fMLP and cytochalasin B (CB) (72).

#### 3.12.2 Lysozyme

Lysozyme hydrolytically splits sugar chains of the peptidoglycan skeleton of bacterial cell wall which leads to the lysis of the bacteria (94). Two research articles investigated the effect of nicotine on the release of lysozyme from neutrophils (64, 73), showing that concentrations previously identified as non-cytotoxic (see above) had no effect, while higher concentrations had contradictory effects.

#### 3.12.3 Myeloperoxidase

MPO catalyzes the reaction of H_2_O_2_ with chloride to generate HOCl within the phagosome. This process leads to the killing of the ingested microorganisms (95, 96). Two studies identified that nicotine had no effect on the release of myeloperoxidase from unstimulated or CB-stimulated neutrophils (72, 73). The same authors reported that nicotine exposure of neutrophils stimulated with both fMLP- and CB decreased the MPO release (72, 73). Loke et al. found that neutrophils pre-treated with very low nicotine concentrations released high levels of MPO when stimulated with calcium ionophore A23187 in the absence and presence of co-pretreatment with cotinine, the predominant metabolite of nicotine (53).

#### 3.12.4 Elastase

Elastase is an antimicrobial protease present in the azurophil granule (96). Five studies investigated the effect of nicotine on neutrophil elastase release (13, 61, 64, 69, 73). Increased release of elastase by unstimulated neutrophils was mainly found when nicotine concentrations in the cytotoxic range were used (73).

## 4 Discussion

This systematic review aimed to analyze the direct impact of nicotine on human neutrophils. Apart from the questions of how chronic nicotine consumption affects immune defense mechanisms, the impact of nicotine on human neutrophils is of high relevance given the importance of smoking for many neutrophil-driven chronic inflammatory diseases such as HS and PPP. Thirty-four research articles were identified that demonstrated analyses of the influence of nicotine on the following neutrophil functions: cell death/damage, apoptosis, chemotaxis, general motility, adhesion molecule expression, eicosanoid synthesis, cytokine/chemokine expression, formation of NETs, phagocytosis, generation of ROS, net antimicrobial activity, and degrading enzyme release.

The research articles that were the basis for this evaluation were mainly conducted in the context of periodontitis, lung injury/metastasis, arthritis, or were in general relation to smoking. Publication years varied from 1977 to 2022. The nicotine concentrations used in the retrieved studies greatly varied, namely between 0.0000005 mmol/l (= 0.5 nmol/l) (67) and 20 mmol/l (49). Effects on neutrophils were actually observed in this broad range of concentrations. The relevance of testing very high concentrations is however limited by two aspects: First, the peak levels reported in individuals were ~0.1 - 500 nmol/l in the blood of smokers (6–11) and ~45 µmol/l in the sputum of vapers (13) and ~30 µmol/l in the sputum of smokers (12). Second, the results compiled in this systematic review suggest that nicotine concentrations above ~3 mmol/l can be considered to be cytotoxic.

**Figure 2:**
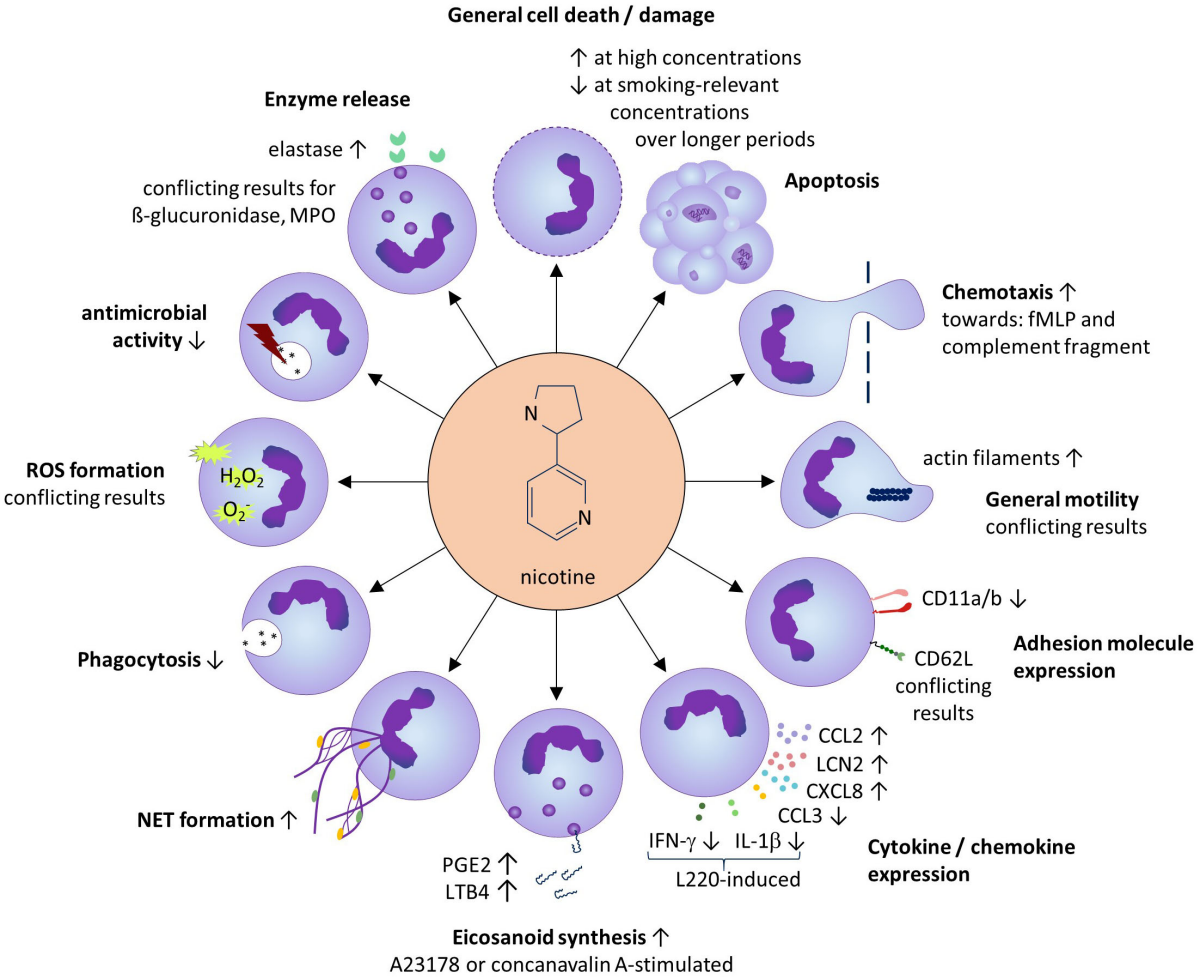
Summarized results of the systematic review. Except for general cell death/damage and apoptosis, the effects of sub-toxic nicotine concentrations on neutrophils are shown.

The results of our study are summarized in **Figure 2** (for references see **Tables 1**–**13**).

Smoking-relevant concentrations applied at longer exposure times (24–72h), supported the neutrophil survival and neutrophil abundance in the tissue. Several studies also demonstrated a strengthening effect of smoking-relevant nicotine concentrations on the neutrophil chemotaxis towards e.g. *N*-formylated tripeptides known to be released by bacteria in the tissue, and a relative increase of the cellular content of filamentous actin, an early indicator of the neutrophil movement that is generated via interconversion of globular actin (97). Interestingly, nicotine also seems to induce the production of chemokines in neutrophils including CXCL8, a ligand of the chemokine receptors CXCR1 and CXCR2 expressed by neutrophils, therefore inducing a positive feedback loop in the attraction of neutrophils to the tissue. As an underlying mechanism, the authors proposed an indirect effect via enhanced peroxinitrit anion (ONOO-) levels, which induced IκB degradation with subsequent activation of NFκB (56).

Apart from the chemotactic attraction, the recruitment of neutrophils from the circulation into the tissue requires their interaction with the activated endothelium of local venules. The current literature did not allow a statement about the influence of nicotine on the neutrophil expression of CD62L. The expression of other neutrophil adhesion molecules such as the integrin α-subunits CD11a and CD11b seems to be negatively influenced by nicotine, while the neutrophil expression of CD18, the β-subunit that forms complexes with these α-subunits, does not seem to be altered by nicotine. As mentioned at the beginning of the paragraph on adhesion molecules, P-selectin glycoprotein ligand 1 is an important adhesion molecule of neutrophils. However, the influence of nicotine on of P-selectin glycoprotein ligand 1 expression has not been investigated in the past and should be targeted in future studies.

Arrived in the tissue, neutrophils play an important role in the early antimicrobial defense. Base on the current literature, the net antimicrobial defense, assessed by the death of several bacteria species after neutrophil encounter, seems to be reduced by nicotine. This might be relevant for infectious diseases as well as for those neutrophil-driven CIDs which are associated with propagation of bacteria in the inflamed tissue, such as HS (98).

The phagocytic uptake of extracellular microbes is the prerequisite for their intracellular killing by the neutrophils. Surprisingly, nicotine impaired the phagocytic uptake of several (opsonized and non-opsonized) particles and bacteria leading to possibly reduced clearance of bacteria. Moreover, due to the highly heterogeneous results found for ROS generation by nicotine-treated neutrophils, no conclusion can be drawn as to whether or not nicotine affects ROS production. We noticed a great variation in the experimental setup among the respective studies, which is known to have a significant impact on the results (99, 100).

Another way neutrophils kill bacteria is the generation of NETs, which consist of decompensated chromatin loaded with cytosolic and granular proteins including antimicrobial proteins (89). The elevation of NET formation by nicotine-exposed neutrophils found in our review also draws the attention back to the CIDs. For instance, NET formation has recently been discovered in the skin lesions of HS patients, where it may support the formation of autoreactive antibodies (101).

Neutrophils are known to support the inflammation by the release of soluble mediators including cytokines/chemokines (102). Surprisingly, the impact of nicotine on cytokine/chemokine release has hardly been studied. The only findings in our review concern CXCL8 (see above) and LCN2, on which nicotine had an inducing influence. However, these results have not been confirmed independently so far. LCN2 also plays a role in HS, where it is released by neutrophils of HS skin lesions (86). Blood LCN2 levels in HS were shown to reflect disease activity (86). LCN2 supports the function and tissue infiltration of neutrophils; in addition, it has direct antimicrobial activity and plays a role in the metabolic control and the induction of inflammatory pain (103), aspects that are all relevant to HS disease (98, 104, 105). Therefore, besides TNF-α (86), nicotine seems to contribute to the elevated LCN2 levels found in HS patients (106).

Interestingly, the only study dealing with the possible impact of nicotine on the production of classical pro- (TNF-α, IL-1β, IFN-γ) and anti-inflammatory (IL-10) cytokines by resting neutrophils did not detect any influence (48). However, in the specific context of infection with parasitic *Entamoeba* species, nicotine was shown to inhibit the L220-induced IL-1β secretion by neutrophils (48).

Activated neutrophils are also important producers of the eicosanoids such as the arachidonic acid metabolites LTB4 and PGE2 (107, 108). Several studies found that nicotine elevated the production of LTB4 by activated neutrophils. LTB4 acts as a strong chemoattractant for neutrophils and is upregulated in HS lesions (109). In HS skin inflammation, PGE2 and LTB4 might also contribute to the pain suffered by those affected (110).

Degrading enzymes found in different types of neutrophilic granules are crucial for the antimicrobial activity of these cells but are also associated with tissue damage in infection and inflammatory settings. The release of only a small range of degrading enzymes have been investigated so far (β-glucuronidase, lysozyme, myeloperoxidase, and elastase), and results only for elastase, showing a nicotine-induced increase, appear to be reliable. Elastase activity is known to play a role in the inflamed tissue in CIDs such as psoriasis, where its activity correlated with disease severity and was diminished after successful treatment (111). The increased elastase activity may also be linked to the nicotine-induced NET formation (112). Further studies are needed to shed light on this interesting connection. Overall, there is little evidence for a general nicotine-induced increase in the tissue-destroying effect of neutrophils.

In the last decade, it has become clear that neutrophils change their expression over their life cycle and in pathological conditions (113). Since in CIDs neutrophils can spend a long time in the inflamed tissue (38, 114, 115), it would be desirable if future studies examined the influence of nicotine on neutrophils at different ages. The neutrophils considered for this systematic review were, as far as stated, from healthy non-smokers. Only two studies, by Srivastava et al. and Murphy et al., investigated the differences in nicotine-induced effect on neutrophils of smokers vs. non-smokers (61, 70). It would be desirable to examine the influence of nicotine on different populations of neutrophils, neutrophils with different activation status, and neutrophils from donors of different ages in future studies. In addition, we see a need for a subsequent systematic review that searches and compiles facts about the influence of nicotine on the production of neutrophil-attracting chemokines by tissue cells and immune cells other than neutrophils. One aspect that has not been investigated in detail in previous studies is the question of whether neutrophils from patients with CIDs react differently to nicotine compared to neutrophils from healthy donors. It is conceivable that the permanent subclinical inflammation that predominates in patients with CIDs (33, 35, 116, 117) alters the activity of neutrophils toward nicotine. This aspect should also be taken into account in future studies.

In conclusion, our systematic literature review only allows a few conclusions regarding the influence of nicotine on the survival and function of neutrophils. Applied at smoking-relevant concentrations, nicotine seems to increase the survival and the chemotaxis of neutrophils towards selected chemoattractants, to elevate their elastase release and their production of mediators with chemoattracting activity (CXCL8, lipocalin-2, LTB4). At the same time, it impairs the phagocytotic and intracellular killing of bacteria by these cells while supporting their NET formation. These aspects are highly relevant to some neutrophil-driven CIDs as described above. In HS, for example, nicotine may support the abundance of neutrophils in the skin, the propagation of bacteria in the presence of those neutrophils, their tissue-degrading activity and the autoimmunity supported by the NET formation. In addition, there are evidences that nicotine induces other HS-relevant effects including epidermal hyperplasia (118) that, however, may be due to nicotine effects on cell types other than neutrophils.

This systematic review has several limitations. The literature search retrieved a multitude of publications with insufficient presentation of the applied methodology necessary for result interpretation. A quality assessment of specific statements on the impact of nicotine on neutrophil function could not be carried out because there is no validated standard for the quality assessment of experimental studies. Moreover, variations in culture conditions among studies could greatly influence neutrophil functions (119). The number of manuscripts investigating specific nicotine effects varied to a great extent (from 1 to 15). Fifteen research articles investigated the impact of nicotine on ROS generation; nevertheless, the contradictory results did not allow any conclusions to be drawn. On the other hand, other aspects (e.g. cytokine/chemokine production and net antimicrobial activity) were only represented by individual studies. In addition, there were large differences in the number of different experimental series dealing with specific nicotine effects and the number of independent experiments carried out per experimental series. To enable the reader to make their own assessment, we have listed all experiment series on a specific nicotine effect individually in each table, and indicated the number of independent experiments performed per series and the level of significance. Finally, the results might be influenced by the general publication bias. In fact, experimental studies with negative results (no effect detected) are less likely to be published (120). As far as it was possible, we displayed all data on the preselected neutrophil biological functions on which no influence of nicotine was discovered.

Hopefully, future studies will reliably help to clarify existing contradictions but also verify the results of individual studies. Moreover, it would be interesting to study the interaction of nicotine with other tobacco components in terms of the neutrophil function.

## Data availability statement

The original contributions presented in the study are included in the article/supplementary material. Further inquiries can be directed to the corresponding author.

## Author contributions

TB: Writing – original draft, Investigation, Visualization, Formal Analysis, Writing – review & editing. RS: Conceptualization, Writing – review & editing, Methodology, Resources. KWi: Writing – review & editing, Investigation, Data curation. TS: Writing – review & editing, Supervision. KWo: Writing – review & editing, Conceptualization, Data curation, Investigation, Supervision, Visualization.

## References

[1] Kozlowski LT Mehta NY Sweeney CT Schwartz SS Vogler GP Jarvis MJ . Filter ventilation and nicotine content of tobacco in cigarettes from Canada, the United Kingdom, and the United States. Tob Control (1998) 7(4):369–75. doi: 10.1136/tc.7.4.369 PMC1751464 10093170

[2] Cunha Pereira R Faria Barbosa W Pereira Lima MA Vieira JOL Jr. Carvalho Guedes RN Rodrigues da Silva BK . Toxicity of botanical extracts and their main constituents on the bees partamona helleri and apis mellifera. Ecotoxicology (2020) 29(3):246–57. doi: 10.1007/s10646-020-02167-7 32170464

[3] Le Foll B Piper ME Fowler CD Tonstad S Bierut L Lu L . Tobacco and nicotine use. Nat Rev Dis Primers (2022) 8(1):19. doi: 10.1038/s41572-022-00346-w 35332148

[4] Cornelius ME Loretan CG Jamal A Davis Lynn BC Mayer M Alcantara IC . Tobacco product use among adults - United States, 2021. MMWR Morb Mortal Wkly Rep (2023) 72(18):475–83. doi: 10.15585/mmwr.mm7218a1 PMC10168602 37141154

[5] Reitsma MB Kendrick P Ababneh E Abbafati C Abbasi-Kangevari M Abdoli A . Spatial, temporal, and demographic patterns in prevalence of smoking tobacco use and attributable disease burden in 204 countries and territories, 1990-2019: A systematic analysis from the global burden of disease study 2019. Lancet (2021) 397(10292):2337–60. doi: 10.1016/s0140-6736(21)01169-7 PMC8223261 34051883

[6] Russell MA Jarvis M Iyer R Feyerabend C . Relation of nicotine yield of cigarettes to blood nicotine concentrations in smokers. Br Med J (1980) 280(6219):972–6. doi: 10.1136/bmj.280.6219.972 PMC1601132 7417765

[7] Hofer I Nil R Wyss F Battig K . The contributions of cigarette yield, consumption, inhalation and puffing behaviour to the prediction of smoke exposure. Clin Investig (1992) 70(3-4):343–51. doi: 10.1007/BF00184671 1521049

[8] Gori GB Lynch CJ . Analytical cigarette yields as predictors of smoke bioavailability. Regul Toxicol Pharmacol (1985) 5(3):314–26. doi: 10.1016/0273-2300(85)90045-5 4059592

[9] Armitage AK Dollery CT George CF Houseman TH Lewis PJ Turner DM . Absorption and metabolism of nicotine from cigarettes. Br Med J (1975) 4(5992):313–6. doi: 10.1136/bmj.4.5992.313 PMC1675218 1192046

[10] Jarvik ME Madsen DC Olmstead RE Iwamoto-Schaap PN Elins JL Benowitz NL . Nicotine blood levels and subjective craving for cigarettes. Pharmacol Biochem Behav (2000) 66(3):553–8. doi: 10.1016/s0091-3057(00)00261-6 10899369

[11] Mello NK Peltier MR Duncanson H . Nicotine levels after iv nicotine and cigarette smoking in men. Exp Clin Psychopharmacol (2013) 21(3):188–95. doi: 10.1037/a0031799 PMC9086919 23647094

[12] Clunes LA Bridges A Alexis N Tarran R . *In vivo* versus in vitro airway surface liquid nicotine levels following cigarette smoke exposure. J Anal Toxicol (2008) 32(3):201–7. doi: 10.1093/jat/32.3.201 PMC2994604 18397571

[13] Ghosh A Coakley RD Ghio AJ Muhlebach MS Esther CR Jr. Alexis NE . Chronic E-cigarette use increases neutrophil elastase and matrix metalloprotease levels in the lung. Am J Respir Crit Care Med (2019) 200(11):1392–401. doi: 10.1164/rccm.201903-0615OC PMC6884043 31390877

[14] Le Foll B Goldberg SR . Effects of nicotine in experimental animals and humans: an update on addictive properties. Handb Exp Pharmacol (2009) 192):335–67. doi: 10.1007/978-3-540-69248-5_12 PMC2687081 19184655

[15] Dani JA De Biasi M . Cellular mechanisms of nicotine addiction. Pharmacol Biochem Behav (2001) 70(4):439–46. doi: 10.1016/s0091-3057(01)00652-9 11796143

[16] Nestler EJ . Is there a common molecular pathway for addiction? Nat Neurosci (2005) 8(11):1445–9. doi: 10.1038/nn1578 16251986

[17] Taly A Corringer PJ Guedin D Lestage P Changeux JP . Nicotinic receptors: allosteric transitions and therapeutic targets in the nervous system. Nat Rev Drug Discovery (2009) 8(9):733–50. doi: 10.1038/nrd2927 19721446

[18] Benhammou K Lee M Strook M Sullivan B Logel J Raschen K . [(3)H]Nicotine binding in peripheral blood cells of smokers is correlated with the number of cigarettes smoked per day. Neuropharmacology (2000) 39(13):2818–29. doi: 10.1016/s0028-3908(00)00153-2 11044752

[19] Vukelic M Qing X Redecha P Koo G Salmon JE . Cholinergic receptors modulate immune complex-induced inflammation in vitro and in vivo. J Immunol (2013) 191(4):1800–7. doi: 10.4049/jimmunol.1203467 23851693

[20] Safronova VG Vulfius CA Shelukhina IV Mal'tseva VN Berezhnov AV Fedotova EI . Nicotinic receptor involvement in regulation of functions of mouse neutrophils from inflammatory site. Immunobiology (2016) 221(7):761–72. doi: 10.1016/j.imbio.2016.01.016 26965141

[21] Takano M Kamei H Nagahiro M Kawami M Yumoto R . Nicotine transport in lung and non-lung epithelial cells. Life Sci (2017) 188:76–82. doi: 10.1016/j.lfs.2017.08.030 28866099

[22] Hecht SS Hatsukami DK . Smokeless tobacco and cigarette smoking: chemical mechanisms and cancer prevention. Nat Rev Cancer (2022) 22(3):143–55. doi: 10.1038/s41568-021-00423-4 PMC9308447 34980891

[23] Hackshaw A Morris JK Boniface S Tang JL Milenković D . Low cigarette consumption and risk of coronary heart disease and stroke: meta-analysis of 141 cohort studies in 55 study reports. Bmj (2018) 360:j5855. doi: 10.1136/bmj.j5855 29367388 PMC5781309

[24] Pietinalho A Pelkonen A Rytilä P . Linkage between smoking and asthma. Allergy (2009) 64(12):1722–7. doi: 10.1111/j.1398-9995.2009.02208.x 19832738

[25] Di Giuseppe D Discacciati A Orsini N Wolk A . Cigarette smoking and risk of rheumatoid arthritis: A dose-response meta-analysis. Arthritis Res Ther (2014) 16(2):R61. doi: 10.1186/ar4498 24594022 PMC4060378

[26] Zhang X Zhang X Yang Y Zhi K Chen Y Zhao J . Association between passive smoking and the risk of rheumatoid arthritis: A systematic review and meta-analysis. Clin Rheumatol (2023) 42(3):663–72. doi: 10.1007/s10067-022-06433-3 36369402

[27] Klareskog L Stolt P Lundberg K Källberg H Bengtsson C Grunewald J . A new model for an etiology of rheumatoid arthritis: smoking may trigger hla-dr (Shared epitope)-restricted immune reactions to autoantigens modified by citrullination. Arthritis Rheum (2006) 54(1):38–46. doi: 10.1002/art.21575 16385494

[28] Wilsmann-Theis D Jacobi A Frambach Y Philipp S Weyergraf A Schill T . Palmoplantar pustulosis - a cross-sectional analysis in Germany. Dermatol Online J (2017) 23(4). doi: 10.5070/D3234034627 28541870

[29] Sabat R Jemec GBE Matusiak Ł Kimball AB Prens E Wolk K . Hidradenitis suppurativa. Nat Rev Dis Primers (2020) 6(1):18. doi: 10.1038/s41572-020-0149-1 32165620

[30] Hutchinson D Shepstone L Moots R Lear JT Lynch MP . Heavy cigarette smoking is strongly associated with rheumatoid arthritis (Ra), particularly in patients without a family history of ra. Ann Rheum Dis (2001) 60(3):223–7. doi: 10.1136/ard.60.3.223 PMC1753588 11171682

[31] Kokolakis G Wolk K Schneider-Burrus S Kalus S Barbus S Gomis-Kleindienst S . Delayed diagnosis of hidradenitis suppurativa and its effect on patients and healthcare system. Dermatology (2020) 236(5):421–30. doi: 10.1159/000508787 PMC7592906 32610312

[32] Sabat R Tsaousi A Ghoreschi K Wolk K Schneider-Burrus S . Sex-disaggregated population analysis in patients with hidradenitis suppurativa. Front Med (Lausanne) (2022) 9:1028943. doi: 10.3389/fmed.2022.1028943 36388895 PMC9663462

[33] Fortes C Mastroeni S Leffondré K Sampogna F Melchi F Mazzotti E . Relationship between smoking and the clinical severity of psoriasis. Arch Dermatol (2005) 141(12):1580–4. doi: 10.1001/archderm.141.12.1580 16365261

[34] Schneider-Burrus S Lux G van der Linde K Barbus S Huss-Marp J Tsaousi A . Hidradenitis suppurativa - prevalence analyses of German statutory health insurance data. J Eur Acad Dermatol Venereol (2021) 35(1):e32–e5. doi: 10.1111/jdv.16783 32580237

[35] Ujiie H Rosmarin D Schon MP Stander S Boch K Metz M . Unmet medical needs in chronic, non-communicable inflammatory skin diseases. Front Med (Lausanne) (2022) 9:875492. doi: 10.3389/fmed.2022.875492 35755063 PMC9218547

[36] Schneider-Burrus S Tsaousi A Barbus S Huss-Marp J Witte K Wolk K . Features associated with quality of life impairment in hidradenitis suppurativa patients. Front Med (Lausanne) (2021) 8:676241. doi: 10.3389/fmed.2021.676241 33987196 PMC8112201

[37] Schneider-Burrus S Kalus S Fritz B Wolk K Gomis-Kleindienst S Sabat R . The impact of hidradenitis suppurativa on professional life. Br J Dermatol (2023) 188(1):122–30. doi: 10.1093/bjd/ljac027 36689513

[38] Tsaousi A Witte E Witte K Röwert-Huber HJ Volk HD Sterry W . Mmp8 is increased in lesions and blood of acne inversa patients: A potential link to skin destruction and metabolic alterations. Mediators Inflammation (2016) 2016:4097574. doi: 10.1155/2016/4097574 PMC5097814 27843200

[39] Wolk K Brembach TC Šimaitė D Bartnik E Cucinotta S Pokrywka A . Activity and components of the granulocyte colony-stimulating factor pathway in hidradenitis suppurativa. Br J Dermatol (2021) 185(1):164–76. doi: 10.1111/bjd.19795 33400270

[40] Wolk K Frambach Y Jacobi A Wilsmann-Theis D Phillipp S Witte-Händel E . Increased levels of lipocalin 2 in palmoplantar pustular psoriasis. J Dermatol Sci (2018) 90(1):68–74. doi: 10.1016/j.jdermsci.2017.12.018 29395573

[41] Sabat R Šimaitė D Gudjonsson JE Brembach TC Witte K Krause T . Neutrophilic granulocyte-derived B-cell activating factor supports B cells in skin lesions in hidradenitis suppurativa. J Allergy Clin Immunol (2023) 151(4):1015–26. doi: 10.1016/j.jaci.2022.10.034 36481267

[42] Ghoreschi K Balato A Enerback C Sabat R . Therapeutics targeting the il-23 and il-17 pathway in psoriasis. Lancet (2021) 397(10275):754–66. doi: 10.1016/S0140-6736(21)00184-7 33515492

[43] Wolk K Wilsmann-Theis D Witte K Brembach TC Kromer C Gerdes S . Interleukin-19 levels are increased in palmoplantar pustulosis and reduced following apremilast treatment. Int J Mol Sci (2023) 24(2):1276–12. doi: 10.3390/ijms24021276 PMC9862858 36674793

[44] Herrero-Cervera A Soehnlein O Kenne E . Neutrophils in chronic inflammatory diseases. Cell Mol Immunol (2022) 19(2):177–91. doi: 10.1038/s41423-021-00832-3 PMC8803838 35039631

[45] Aspera-Werz RH Mück J Linnemann C Herbst M Ihle C Histing T . Nicotine and cotinine induce neutrophil extracellular trap formation-potential risk for impaired wound healing in smokers. Antioxidants (Basel) (2022) 11(12):2424–14. doi: 10.3390/antiox11122424 PMC9774423 36552632

[46] Tyagi A Sharma S Wu K Wu SY Xing F Liu Y . Nicotine promotes breast cancer metastasis by stimulating N2 neutrophils and generating pre-metastatic niche in lung. Nat Commun (2021) 12(1):474. doi: 10.1038/s41467-020-20733-9 33473115 PMC7817836

[47] White PC Hirschfeld J Milward MR Cooper PR Wright HJ Matthews JB . Cigarette smoke modifies neutrophil chemotaxis, neutrophil extracellular trap formation and inflammatory response-related gene expression. J Periodontal Res (2018) 53(4):525–35. doi: 10.1111/jre.12542 29574730

[48] Villalobos-Gomez FD Garcia-Lorenzane M Escobedo G Talamas-Rohana P Salinas-Gutierrez R Hernandez-Ramirez VI . Entamoeba histolytica L220 induces the in vitro activation of macrophages and neutrophils and is modulated by neurotransmitters. Acta Parasitologica (2018) 63(2):270–9. doi: 10.1515/ap-2018-0031 29654688

[49] Lee J Luria A Rhodes C Raghu H Lingampalli N Sharpe O . Nicotine drives neutrophil extracellular traps formation and accelerates collagen-induced arthritis. Rheumatol (Oxford) (2017) 56(4):644–53. doi: 10.1093/rheumatology/kew449 PMC5850711 28013195

[50] Hosseinzadeh A Thompson PR Segal BH Urban CF . Nicotine induces neutrophil extracellular traps. J Leukoc Biol (2016) 100(5):1105–12. doi: 10.1189/jlb.3AB0815-379RR PMC5069087 27312847

[51] Xu Y Li H Bajrami B Kwak H Cao S Liu P . Cigarette smoke (Cs) and nicotine delay neutrophil spontaneous death *via* suppressing production of diphosphoinositol pentakisphosphate. Proc Natl Acad Sci U.S.A. (2013) 110(19):7726–31. doi: 10.1073/pnas.1302906110 PMC3651495 23610437

[52] Matthews JB Chen FM Milward MR Ling MR Chapple IL . Neutrophil superoxide production in the presence of cigarette smoke extract, nicotine and cotinine. J Clin Periodontol (2012) 39(7):626–34. doi: 10.1111/j.1600-051X.2012.01894.x 22607095

[53] Loke WM Lam KM Chong WL Chew SE Quek AM Lim E . Products of 5-lipoxygenase and myeloperoxidase activities are increased in young male cigarette smokers. Free Radic Res (2012) 46(10):1230–7. doi: 10.3109/10715762.2012.701291 22690830

[54] Matthews JB Chen FM Milward MR Wright HJ Carter K McDonagh A . Effect of nicotine, cotinine and cigarette smoke extract on the neutrophil respiratory burst. J Clin Periodontol (2011) 38(3):208–18. doi: 10.1111/j.1600-051X.2010.01676.x 21214612

[55] Al-Shibani NK Labban NY Kowolik MJ Ruby JD Windsor LJ . Responses of human neutrophils to nicotine and/or porphyromonas gingivalis. J Periodontol (2011) 82(10):1504–8. doi: 10.1902/jop.2011.100757 21342004

[56] Iho S Tanaka Y Takauji R Kobayashi C Muramatsu I Iwasaki H . Nicotine induces human neutrophils to produce il-8 through the generation of peroxynitrite and subsequent activation of nf-kappab. J Leukoc Biol (2003) 74(5):942–51. doi: 10.1189/jlb.1202626 12960242

[57] Speer P Zhang Y Gu Y Lucas MJ Wang Y . Effects of nicotine on intercellular adhesion molecule expression in endothelial cells and integrin expression in neutrophils in vitro. Am J Obstet Gynecol (2002) 186(3):551–6. doi: 10.1067/mob.2002.121106 11904622

[58] Mariggiò MA Guida L Laforgia A Santacroce R Curci E Montemurro P . Nicotine effects on polymorphonuclear cell apoptosis and lipopolysaccharide-induced monocyte functions. A Possible Role Periodontal Disease? J Periodontal Res (2001) 36(1):32–9. doi: 10.1034/j.1600-0765.2001.00301.x 11246702

[59] Yoshida H Sakagami H Yamanaka Y Amano Y Yamaguchi M Yamamura M . Induction of DNA fragmentation by nicotine in human myelogenous leukemic cell lines. Anticancer Res (1998) 18(4a):2507–11. 9703901

[60] Ryder MI Fujitaki R Lebus S Mahboub M Faia B Muhaimin D . Alterations of neutrophil L-selectin and cd18 expression by tobacco smoke: implications for periodontal diseases. J Periodontal Res (1998) 33(6):359–68. doi: 10.1111/j.1600-0765.1998.tb02211.x 9777587

[61] Murphy EA Danna-Lopes D Sarfati I Rao SK Cohen JR . Nicotine-stimulated elastase activity release by neutrophils in patients with abdominal aortic aneurysms. Ann Vasc Surg (1998) 12(1):41–5. doi: 10.1007/s100169900113 9451995

[62] Aoshiba K Nagai A Yasui S Konno K . Nicotine prolongs neutrophil survival by suppressing apoptosis. J Lab Clin Med (1996) 127(2):186–94. doi: 10.1016/s0022-2143(96)90077-3 8636647

[63] Pabst MJ Pabst KM Collier JA Coleman TC Lemons-Prince ML Godat MS . Inhibition of neutrophil and monocyte defensive functions by nicotine. J Periodontol (1995) 66(12):1047–55. doi: 10.1902/jop.1995.66.12.1047 8683417

[64] Seow WK Thong YH Nelson RD MacFarlane GD Herzberg MC . Nicotine-induced release of elastase and eicosanoids by human neutrophils. Inflammation (1994) 18(2):119–27. doi: 10.1007/bf01534553 8070897

[65] Ryder MI . Nicotine effects on neutrophil F-actin formation and calcium release: implications for tobacco use and pulmonary diseases. Exp Lung Res (1994) 20(4):283–96. doi: 10.3109/01902149409064388 7988493

[66] Aoshiba K Nagai A Konno K . Nicotine prevents a reduction in neutrophil filterability induced by cigarette smoke exposure. Am J Respir Crit Care Med (1994) 150(4):1101–7. doi: 10.1164/ajrccm.150.4.7921443 7921443

[67] Saareks V Riutta A Mucha I Alanko J Vapaatalo H . Nicotine and cotinine modulate eicosanoid production in human leukocytes and platelet rich plasma. Eur J Pharmacol (1993) 248(4):345–9. doi: 10.1016/0926-6917(93)90012-f 8181543

[68] Nowak D Ruta U Piasecka G . Nicotine increases human polymorphonuclear leukocytes chemotactic response–a possible additional mechanism of lung injury in cigarette smokers. Exp Pathol (1990) 39(1):37–43. doi: 10.1016/s0232-1513(11)80218-5 2394238

[69] Nowak D Ruta U . Nicotine inhibits alpha-1-proteinase inhibitor inactivation by oxidants derived from human polymorphonuclear leukocytes. Exp Pathol (1990) 38(4):249–55. doi: 10.1016/s0232-1513(11)80236-7 2167236

[70] Srivastava ED Hallett MB Rhodes J . Effect of nicotine and cotinine on the production of oxygen free radicals by neutrophils in smokers and non-smokers. Hum Toxicol (1989) 8(6):461–3. doi: 10.1177/096032718900800605 2591986

[71] Jay M Kojima S Gillespie MN . Nicotine potentiates superoxide anion generation by human neutrophils. Toxicol Appl Pharmacol (1986) 86(3):484–7. doi: 10.1016/0041-008x(86)90376-5 3024359

[72] Sasagawa S Suzuki K Sakatani T Fujikura T . Effects of nicotine on the functions of human polymorphonuclear leukocytes in vitro. J Leukoc Biol (1985) 37(5):493–502. doi: 10.1002/jlb.37.5.493 2984301

[73] Totti N 3rd McCusker KT Campbell EJ Griffin GL Senior RM . Nicotine is chemotactic for neutrophils and enhances neutrophil responsiveness to chemotactic peptides. Science (1984) 223(4632):169–71. doi: 10.1126/science.6318317 6318317

[74] Sasagawa S Kameda H Sudo J Tanabe T . Inhibitory effect of nicotine on chemiluminescence response of human polymorphonuclear leukocytes stimulated by opsonized zymosan in vitro. J Toxicol Sci (1984) 9(1):1–9. doi: 10.2131/jts.9.1 6471124

[75] Gala D Kreilick RW Hoss W Matchett S . Nicotine-induced membrane perturbation of intact human granulocytes spin-labeled with 5-doxylstearic acid. Correlation with chemotaxis. Biochim Biophys Acta (1984) 778(3):503–10. doi: 10.1016/0005-2736(84)90400-0 6095910

[76] Bridges RB Kraal JH Huang LJ Chancellor MB . Effects of cigarette smoke components on in vitro chemotaxis of human polymorphonuclear leukocytes. Infect Immun (1977) 16(1):240–8. doi: 10.1128/iai.16.1.240-248.1977 PMC421514 873608

[77] Noseykina EM Schepetkin IA Atochin DN . Molecular mechanisms for regulation of neutrophil apoptosis under normal and pathological conditions. J Evol Biochem Physiol (2021) 57(3):429–50. doi: 10.1134/s0022093021030017 PMC8245921 34226754

[78] Liles WC Kiener PA Ledbetter JA Aruffo A Klebanoff SJ . Differential expression of fas (Cd95) and fas ligand on normal human phagocytes: implications for the regulation of apoptosis in neutrophils. J Exp Med (1996) 184(2):429–40. doi: 10.1084/jem.184.2.429 PMC2192712 8760796

[79] Capucetti A Albano F Bonecchi R . Multiple roles for chemokines in neutrophil biology. Front Immunol (2020) 11:1259. doi: 10.3389/fimmu.2020.01259 32733442 PMC7363767

[80] Fritz-Laylin LK Riel-Mehan M Chen BC Lord SJ Goddard TD Ferrin TE . Actin-based protrusions of migrating neutrophils are intrinsically lamellar and facilitate direction changes. Elife (2017) 6. doi: 10.7554/eLife.26990 PMC5614560 28948912

[81] Cappenberg A Kardell M Zarbock A . Selectin-mediated signaling-shedding light on the regulation of integrin activity in neutrophils. Cells (2022) 11(8):1310–18. doi: 10.3390/cells11081310 PMC9025114 35455989

[82] Sheppe AEF Edelmann MJ . Roles of eicosanoids in regulating inflammation and neutrophil migration as an innate host response to bacterial infections. Infect Immun (2021) 89(8):e0009521. doi: 10.1128/iai.00095-21 34031130 PMC8281227

[83] Tsai CY Hsieh SC Liu CW Lu CS Wu CH Liao HT . Cross-talk among polymorphonuclear neutrophils, immune, and non-immune cells *via* released cytokines, granule proteins, microvesicles, and neutrophil extracellular trap formation: A novel concept of biology and pathobiology for neutrophils. Int J Mol Sci (2021) 22(6):3119–27. doi: 10.3390/ijms22063119 PMC8003289 33803773

[84] González LA Melo-González F Sebastián VP Vallejos OP Noguera LP Suazo ID . Characterization of the anti-inflammatory capacity of il-10-producing neutrophils in response to streptococcus pneumoniae infection. Front Immunol (2021) 12:638917. doi: 10.3389/fimmu.2021.638917 33995357 PMC8113954

[85] Witte-Händel E Wolk K Tsaousi A Irmer ML Mößner R Shomroni O . The il-1 pathway is hyperactive in hidradenitis suppurativa and contributes to skin infiltration and destruction. J Invest Dermatol (2019) 139(6):1294–305. doi: 10.1016/j.jid.2018.11.018 30528824

[86] Wolk K Wenzel J Tsaousi A Witte-Händel E Babel N Zelenak C . Lipocalin-2 is expressed by activated granulocytes and keratinocytes in affected skin and reflects disease activity in acne inversa/hidradenitis suppurativa. Br J Dermatol (2017) 177(5):1385–93. doi: 10.1111/bjd.15424 28256718

[87] Lehmann MH Torres-Domínguez LE Price PJ Brandmüller C Kirschning CJ Sutter G . Ccl2 expression is mediated by type I ifn receptor and recruits nk and T cells to the lung during mva infection. J Leukoc Biol (2016) 99(6):1057–64. doi: 10.1189/jlb.4MA0815-376RR 26992431

[88] Dommel S Blüher M . Does C-C motif chemokine ligand 2 (Ccl2) link obesity to a pro-inflammatory state? Int J Mol Sci (2021) 22(3):1500–20. doi: 10.3390/ijms22031500 PMC7867366 33540898

[89] Vorobjeva NV Chernyak BV . Netosis: molecular mechanisms, role in physiology and pathology. Biochem (Mosc) (2020) 85(10):1178–90. doi: 10.1134/s0006297920100065 PMC7590568 33202203

[90] Burn GL Foti A Marsman G Patel DF Zychlinsky A . The neutrophil. Immunity (2021) 54(7):1377–91. doi: 10.1016/j.immuni.2021.06.006 34260886

[91] Winterbourn CC Kettle AJ Hampton MB . Reactive oxygen species and neutrophil function. Annu Rev Biochem (2016) 85:765–92. doi: 10.1146/annurev-biochem-060815-014442 27050287

[92] Rister M Nett U . Beta-glucuronidase release from leukocytes in children. Klin Wochenschr (1985) 63(12):540–4. doi: 10.1007/bf01733198 3861904

[93] Paigen K . Mammalian beta-glucuronidase: genetics, molecular biology, and cell biology. Prog Nucleic Acid Res Mol Biol (1989) 37:155–205. doi: 10.1016/s0079-6603(08)60698-4 2672109

[94] Ragland SA Criss AK . From bacterial killing to immune modulation: recent insights into the functions of lysozyme. PloS Pathog (2017) 13(9):e1006512. doi: 10.1371/journal.ppat.1006512 28934357 PMC5608400

[95] Nauseef WM . Myeloperoxidase in human neutrophil host defence. Cell Microbiol (2014) 16(8):1146–55. doi: 10.1111/cmi.12312 PMC4301731 24844117

[96] Uribe-Querol E Rosales C . Control of phagocytosis by microbial pathogens. Front Immunol (2017) 8:1368. doi: 10.3389/fimmu.2017.01368 29114249 PMC5660709

[97] Saiepour D Sehlin J Oldenborg PA . Insulin inhibits phagocytosis in normal human neutrophils *via* pkcalpha/beta-dependent priming of F-actin assembly. Inflammation Res (2006) 55(3):85–91. doi: 10.1007/s00011-005-0009-1 16673150

[98] Wolk K Join-Lambert O Sabat R . Aetiology and pathogenesis of hidradenitis suppurativa. Br J Dermatol (2020) 183(6):999–1010. doi: 10.1111/bjd.19556 33048349

[99] Gomes A Fernandes E Lima JL . Fluorescence probes used for detection of reactive oxygen species. J Biochem Biophys Methods (2005) 65(2-3):45–80. doi: 10.1016/j.jbbm.2005.10.003 16297980

[100] Pavelescu LA . On reactive oxygen species measurement in living systems. J Med Life (2015) 8 Spec Issue(Spec Issue):38–42. PMC4564046 26361509

[101] Byrd AS Carmona-Rivera C O'Neil LJ Carlucci PM Cisar C Rosenberg AZ . and type I interferons contribute to immune dysregulation in hidradenitis suppurativa. Sci Transl Med (2019) 11(508):eaav5908. doi: 10.1126/scitranslmed.aav5908 31484788 PMC11369904

[102] Tecchio C Micheletti A Cassatella MA . Neutrophil-derived cytokines: facts beyond expression. Front Immunol (2014) 5:508. doi: 10.3389/fimmu.2014.00508 25374568 PMC4204637

[103] Chakraborty S Kaur S Guha S Batra SK . The multifaceted roles of neutrophil gelatinase associated lipocalin (Ngal) in inflammation and cancer. Biochim Biophys Acta (2012) 1826(1):129–69. doi: 10.1016/j.bbcan.2012.03.008 PMC3362670 22513004

[104] Sabat R Chanwangpong A Schneider-Burrus S Metternich D Kokolakis G Kurek A . Increased prevalence of metabolic syndrome in patients with acne inversa. PloS One (2012) 7(2):e31810. doi: 10.1371/journal.pone.0031810 22359634 PMC3281019

[105] von der Werth JM Jemec GB . Morbidity in patients with hidradenitis suppurativa. Br J Dermatol (2001) 144(4):809–13. doi: 10.1046/j.1365-2133.2001.04137.x 11298541

[106] Abella V Scotece M Conde J Gómez R Lois A Pino J . The potential of lipocalin-2/ngal as biomarker for inflammatory and metabolic diseases. Biomarkers (2015) 20(8):565–71. doi: 10.3109/1354750x.2015.1123354 PMC4819811 26671823

[107] Shindo K Koide K Fukumura M . Enhancement of leukotriene B4 release in stimulated asthmatic neutrophils by platelet activating factor. Thorax (1997) 52(12):1024–9. doi: 10.1136/thx.52.12.1024 PMC1758460 9516893

[108] Ford-Hutchinson AW Bray MA Doig MV Shipley ME Smith MJ . Leukotriene B, a potent chemokinetic and aggregating substance released from polymorphonuclear leukocytes. Nature (1980) 286(5770):264–5. doi: 10.1038/286264a0 6250050

[109] Penno CA Jager P Laguerre C Hasler F Hofmann A Gass SK . Lipidomics profiling of hidradenitis suppurativa skin lesions reveals lipoxygenase pathway dysregulation and accumulation of proinflammatory leukotriene B4. J Invest Dermatol (2020) 140(12):2421–32 e10. doi: 10.1016/j.jid.2020.04.011 32387270

[110] Inazumi T Yamada K Shirata N Sato H Taketomi Y Morita K . Prostaglandin E(2)-ep4 axis promotes lipolysis and fibrosis in adipose tissue leading to ectopic fat deposition and insulin resistance. Cell Rep (2020) 33(2):108265. doi: 10.1016/j.celrep.2020.108265 33053354

[111] Wiedow O Wiese F Christophers E . Lesional elastase activity in psoriasis. Diagnostic and prognostic significance. Arch Dermatol Res (1995) 287(7):632–5. doi: 10.1007/bf00371734 8534125

[112] Papayannopoulos V Metzler KD Hakkim A Zychlinsky A . Neutrophil elastase and myeloperoxidase regulate the formation of neutrophil extracellular traps. J Cell Biol (2010) 191(3):677–91. doi: 10.1083/jcb.201006052 PMC3003309 20974816

[113] Silvestre-Roig C Fridlender ZG Glogauer M Scapini P . Neutrophil diversity in health and disease. Trends Immunol (2019) 40(7):565–83. doi: 10.1016/j.it.2019.04.012 PMC7185435 31160207

[114] Kolaczkowska E Kubes P . Neutrophil recruitment and function in health and inflammation. Nat Rev Immunol (2013) 13(3):159–75. doi: 10.1038/nri3399 23435331

[115] Colotta F Re F Polentarutti N Sozzani S Mantovani A . Modulation of granulocyte survival and programmed cell death by cytokines and bacterial products. Blood (1992) 80(8):2012–20. doi: 10.1182/blood.V80.8.2012.2012 1382715

[116] Popescu D Rezus E Badescu MC Dima N Seritean Isac PN Dragoi IT . Cardiovascular risk assessment in rheumatoid arthritis: accelerated atherosclerosis, new biomarkers, and the effects of biological therapy. Life (Basel) (2023) 13(2):319–26. doi: 10.3390/life13020319 PMC9965162 36836675

[117] Sabat R Gudjonsson JE Brembilla NC van Straalen KR Wolk K . Biology of interleukin-17 and novel therapies for hidradenitis suppurativa. J Interferon Cytokine Res (2023) 43. doi: 10.1089/jir.2023.0105 37824200

[118] Hana A Booken D Henrich C Gratchev A Maas-Szabowski N Goerdt S . Functional significance of non-neuronal acetylcholine in skin epithelia. Life Sci (2007) 80(24-25):2214–20. doi: 10.1016/j.lfs.2007.02.007 17363005

[119] Blanter M Gouwy M Struyf S . Studying neutrophil function in vitro: cell models and environmental factors. J Inflammation Res (2021) 14:141–62. doi: 10.2147/jir.S284941 PMC7829132 33505167

[120] Echevarría L Malerba A Arechavala-Gomeza V . Researcher's perceptions on publishing "Negative" Results and open access. Nucleic Acid Ther (2021) 31(3):185–9. doi: 10.1089/nat.2020.0865 PMC8215407 32730128

